# *Hibberdia magna* (Chrysophyceae): a promising freshwater fucoxanthin and polyunsaturated fatty acid producer

**DOI:** 10.1186/s12934-023-02061-x

**Published:** 2023-04-19

**Authors:** Antonín Střížek, Pavel Přibyl, Martin Lukeš, Tomáš Grivalský, Jiří Kopecký, Tomáš Galica, Pavel Hrouzek

**Affiliations:** 1 Laboratory of Algal Biotechnology, Institute of Microbiology of the Czech Academy of Sciences - Center Algatech, Trebon, Czech Republic; 2 Centre for Phycology, Institute of Botany of the Czech Academy of Sciences, Trebon, Czech Republic; 3 Department of Ecology, Faculty of Science, Charles University, Prague, Czech Republic

**Keywords:** Microalgae, Fucoxanthin, Chrysophyceae, *Hibberdia magna*, Polyunsaturated fatty acids, Volumetric productivity, Light and temperature effect

## Abstract

**Background:**

Algae are prominent producers of carotenoids and polyunsaturated fatty acids which are greatly prized in the food and pharmaceutic industry. Fucoxanthin represents a notable high-value carotenoid produced exclusively by algae. Its benefits range far beyond just antioxidant activity and include cancer prevention, anti-diabetes, anti-obesity, and many other positive effects. Accordingly, large-scale microalgae cultivation to produce fucoxanthin and polyunsaturated fatty acids is still under intensive development in the commercial and academic sectors. Industrially exploitable strains are predominantly derived from marine species while comparable freshwater fucoxanthin producers have yet to be explored.

**Results:**

In this study, we searched for freshwater fucoxanthin producers among photoautotrophic flagellates including members of the class Chrysophyceae. The initial screening turned our attention to the chrysophyte alga *Hibberdia magna*. We performed a comprehensive cultivation experiments using a temperature × light cross-gradient to assess the impact of these conditions on the target compounds productivity. Here we present the observations that* H*. *magna* simultaneously produces fucoxanthin (max. 1.2% dry biomass) and polyunsaturated fatty acids (max. ~ 9.9% dry biomass) and is accessible to routine cultivation in lab-scale conditions. The highest biomass yields were 3.73 g L^−1^ accompanied by maximal volumetric productivity of 0.54 g L^−1^ d^−1^ which are comparable values to marine microalgae fucoxanthin producers in phototrophic mode. *H. magna* demonstrated different optimal conditions for biomass, fucoxanthin, and fatty acid accumulation. While maximal fucoxanthin productivities were obtained in dim light and moderate temperatures (23 °C× 80 µmol m^−2^ s^−1^), the highest PUFA and overall biomass productivities were found in low temperature and high light (17–20 °C × 320–480 µmol m^−2^ s^−1^). Thus, a smart biotechnology setup should be designed to fully utilize *H*. *magna* biotechnological potential.

**Conclusions:**

Our research brings pioneer insight into the biotechnology potential of freshwater autotrophic flagellates and highlights their ability to produce high-value compounds. Freshwater fucoxanthin-producing species are of special importance as the use of sea-water-based media may increase cultivation costs and prohibits inland microalgae production.

**Supplementary Information:**

The online version contains supplementary material available at 10.1186/s12934-023-02061-x.

## Background

A feasible technology to produce microalgal biomass in bulk is still far from being achieved despite decades of intensive research. Hence, recent algal biotechnological aims have been shifting from bulk commodities to algae-specific fine products with high commercial value. Some of these high-value products are carotenoids and polyunsaturated fatty acids (PUFA) which both demonstrate human health benefits [1, 2] and also a broad application in the aquaculture and feed industry [3] leading to a rapidly growing demand.

Fucoxanthin (FX), a carotenoid pigment classified among xanthophylls, is the most abundant carotenoid accounting for approximately 10% of all carotenoids [4] on the Earth and plays a role in light acquisition, predominantly in Ochrophyta and Haptophyta [5]. It is an algae-specific product, without artificial substitutes so far [6]. It has many positive effects on human health and interest in its applications is considerably growing. According to Pajot et al. [7], 28 peer-reviewed research papers (published between 2020 and 2021) refer to FX anti-cancer, anti-inflammatory, anti-obesity, anti-diabetes, anti-Alzheimer, antioxidant, generally protective and other positive effects just in the past two years. Its bioactivity and physiological effects are suggested to be caused by its unique structure in polyene chromophore which is an allenic bond and two hydroxyl groups [8]. Nowadays, seaweed (Phaeophyceae), a traditional part of the seafood diet, is the major source of FX for human consumption. However, for inland countries, seaweeds are usually rarely accessible and generally unpopular. Moreover, FX content in seaweed biomass is low in concentration [9], poorly bio-available [10], and difficult to extract [11]. Hence the novel source of this carotenoid is under intensive investigation and microalgae from other Ochrophyta taxa could be a promising alternative. Advanced microalgae cultivation technologies and precisely optimized species and site-specific culture protocols can effectively boost FX productivity [12].

The situation with PUFA production by microalgae is quite different compared to FX. PUFA are not algae-specific compounds, as they are also produced by other protist groups and vascular plants [13]. However, most of the commonly consumed foods from plants and the meat of farm animals possess just limited PUFA content and their Omega 6 to Omega 3 PUFA ratio is not appropriate for a healthy human diet [14]. Whereas it is known that PUFA are essential in the diet of all animals including humans, there is convincing evidence that only Omega 6 Linoleic acid (LA, 18:2ω6) and Omega 3 Alpha-linolenic acid (ALA, 18:3ω3) are truly essential as they can’t be synthesized by humans [15]. Other PUFA are transformed from LA and ALA, but their output is limited by the low capacity of the enzymatic apparatus of human elongases and desaturases [16]. Hence the long-chain PUFA (LC-PUFA) such as eicosapentaenoic acid (EPA, 20:5ω3) or docosahexaenoic acid (DHA, 22:6ω3) are considered to be conditionally essential and they are needed in higher amounts mostly during prenatal and early child development periods [1]. LC-PUFA intake is beneficial for a healthy lifestyle and is generally recommended by nutritionists [1]. Although there are several sources of LC-PUFA for human consumption (mostly fish and other seafood), microalgae are primary producers of PUFA and LC-PUFA in the vast majority of aquatic habitats hence they present a suitable and especially sustainable source of these compounds [17].

The interconnected effects of the growing human population and extensive damage to aquatic ecosystems caused by over-fishing calls for innovative solutions that will help to reduce the dependence on the traditional ways of acquiring PUFA and FX. A recent approach to the combined production of PUFA and FX by microalgae dominantly relies on marine algae species belonging to marine diatoms or Haptophyceae [18, 19]. Only a very limited number of studies have investigated freshwater microalgae from this point of view, despite microalgae cultivation in artificial conditions in seawater-based media brings several disadvantages. Most land is distant from the coast so the utilization of filtered seawater is either not possible or extremely expensive. The inland preparation of artificial seawater is complicated and costly, and due to its subsequent loss through regular wastewater treatment plants can be applied only in low amounts, so a technically demanding recycling system for cultivation medium needs to be put in place [20]. In addition, cultivation systems and downstream processing facilities must be resistant to the corrosive effects of saltwater. Freshwater algae cultivation is more universally applicable although the usual complications of microalgae cultivation persist. A deeper investigation of the feasibility of freshwater FX and PUFA microalgae production is well worth being evaluated.

Despite freshwater photoautotrophic flagellates being important and common members of limnetic communities and having a great potential to produce high-value compounds, they remain a highly understudied group in the context of applied phycology. Unlike also very common freshwater diatoms, they are not to such a high degree dependent on sexual reproduction, and hence can be kept in monoclonal cultures more easily, and do not necessarily constitute extracellular siliceous structures as diatoms do. Through the present study, we want to establish the base point for further research focused on the freshwater photoautotrophic flagellates as prospective FX and PUFA producers and to provide an alternative to the present-day FX production exclusively from marine microalgae. For the intended goal, we performed screening of FX-producing freshwater autotrophic flagellate strains achievable from culture collections, mostly focused on chrysophytes, and for further work, we selected the best performing organism *Hibberdia magna.*

*Hibberdia magna* (Belcher) Andersen (1989) is a newly examined strain in the sense of applied microalgal research. It was identified and isolated from a small pond near the city of Cambridge (England, UK) (52°08′51′′N, 000°03′12′′W) in 1972 by J.H. Belcher, who described it as *Chrysosphaera magna* [21]. Since then, it has been maintained at CCMP (Culture Collection of Marine Phytoplankton, USA) and later at NCMA culture collections (Provasoli-Guillard National Center for Marine Algae and Microbiota, USA). In 1986 R.A. Andersen renamed it due to its unique orientation of the flagellar apparatus as a holotype species: *Hibberdia magna* [22], and this taxonomical value was lately confirmed by the phylogeny analysis of the SSU rRNA [23, 24]. To the best of our knowledge, this species has not been reported to be found in nature since then. This original strain is currently maintained in four large algal culture collections (UTEX, CCAP, NORCCA, and NCMA) at least. Each of the culture collections use slightly different culture media, temperature, and light conditions, proving this strain is relatively easy to maintain in vivo and accessible for experimental work.

In the present study, we performed comprehensive manipulation cultivation experiments focused on the growth performances of *H. magna* as a candidate organism for combined FX and PUFA production.

## Results and discussion

The large-scale microalgae cultivation for FX production is still under intensive development in both the commercial and academic sectors. So far, the technology employing only a few marine algae species belonging exclusively to diatoms and haptophytes has been established [12]. In addition, there are very few records on freshwater FX producers, even though diatoms and chrysophytes are very abundant and diverse in most freshwater habitats. Petrushkina et al. [25] cultured and analyzed several freshwater diatoms, and one chrysophyte alga *Mallomonas kalinae* for FX content. Gérin et al. [26] optimized culture media for two species of freshwater diatoms *Sellaphora minima* and *Nitzschia palea*. Some freshwater members of phago-mixotrophic chrysophyte genera *Ochromonas* and *Poterioochromonas* were analyzed for FX content [27, 28]. The investigation of freshwater photoautotrophic chrysophytes as a source of PUFA is also a rare research topic. Klaveness [29] reported the filamentous colonial alga *Hydrurus foetidus* as a potential producer of LC-PUFA and Wacker et al. [30] tested the impact of different light intensities on the FA composition of the *Chromulina* sp. In addition to these records, Ruffell et al. [31] characterized the simultaneous production of FX and PUFA by the marine chrysophyte *Boekelovia hooglandii* for its potential in aquaculture.

### Strain selection

Motivated by this lack of exploration of the freshwater algae for its simultaneous FX and PUFA production we selected 10 strains from the world culture collections. These were selected based on several criteria (see Methods - Algal strain screening) to indicate the potential that these strains may have for applied biomass production (Table 1). Unfortunately, only 5 of the obtained strains were cultivable after transport. Initially, we performed several growth tests and biomass content analyses of the target products to select the most suitable one for further research.

**Table 1:** Strains obtained from algal culture collections, their performance during pilot growing tests and FX/PUFA content analysis represented as lowest and highest observed values

Organism	Culture Collection	Strain code	Viable	Dens. Max [DWg L^−1^]	FX [mg DWg^−1^]		PUFA [mg DWg^−1^]		LC-PUFA [mg DWg^−1^]	
					lowest	highest	lowest	highest	lowest	highest
*Incertae sedis*										
*Tetrasporopsis sp.*	SAG	20.88	no	–	–	–	–	–	–	–
Chrysophyceae										
*Chromulina nebulosa*	CCAC	4405 b	no	–	–	–	–	–	–	–
*Chromulina nebulosa*	NORCCA	K-1162	no	–	–	-	–	–	–	–
*Chromulina nebulosa*	CCAC	4411 b	no	–	–	-	–	–	–	–
*Mallomonas striata*	CCMP	2059	no	–	–	-	–	–	–	–
*Mallomonas rasilis*	NORCCA	K-1180	yes	0.16	2.19	4.96	**47.50**	62.15	3.97	21.11
*Chrysosaccus sp.I*	CCAC	3965 b	yes	0.56	2.19	6.00	25.18	78.97	8.44	21.33
*Chrysosaccus sp.II*	NORCCA	K-1204	yes	0.36	2.50	7.05	17.43	47.51	7.51	11.42
*Hibberdia magna*	NORCCA	K-1175	yes	0.64	**2.70**	**13.25**	22.54	**82.56**	9.62	26.76
Pavlovophyceae										
*Diacronema noctivaga*	SAG	5.83	yes	**2.57**	0.15	3.47	27.95	56.87	**19.74**	**37.68**

Bold—the highest observed values among strains

The first considered organism was *Diacronema noctivaga* (formerly gen. *Pavlova*) a rare freshwater representative of the class Pavlovophyceae (Haptophyta). Some marine species of the genus *Diacronema* are utilized in bivalve hatcheries [32], hence we expected this species could be a good candidate for further research. During pilot cultivations, *D. noctivaga* demonstrated the highest DW densities and the highest LC-PUFA content per DW. However, its FX content was low in comparison with other candidates (Table 1). The pilot growth tests (data not shown) revealed certain difficulties in its life cycle. When it was in the palmelloid stage (immobile aggregated individuals) it performed significant growth, but after transforming into flagellates it stopped growing. We were not able to induce the reverse transition from flagellates to the palmelloid stage and to maintain consistent growth of this algal strain.

*Hibberdia magna* produced the highest values of all monitored parameters among other chrysophytes (Table 1) including a very high FX content and its growth was solid and consistent. The organism did not exhibit complex life cycle and only flagellate stage was observed. After optimization of culture media, it also grew to the highest biomass densities and hence was chosen for further exploration.

### Temperature light cross-gradient

#### General results of cross-gradient

The cross-gradient experiment aimed to identify the optimal conditions to produce *Hibberdia magna* biomass with a focus on enhanced content of desired high-value products, namely FX and Omega3 and Omega6 PUFA. For this purpose, a narrowed range of temperature and light intensities was selected appropriately to define production optima for DW, FX, or FA, as shown further. Generally, border conditions of selected gradients did not support optimal biomass growth or target compound production. The highest selected temperature (26 °C) was borderline for survival and at the lowest temperature (14 °C) the growth rates were considerably suppressed. Similarly, the highest light intensity of 640 µmol m^−2^ s^−1^ was stressful for the organism and, in combination with low temperature, represented lethal conditions. The lowest light intensity of 40 µmol m^−2^ s^−1^ reduced the growth rates.

#### Variability among experiment replications

**Fig. 1:**
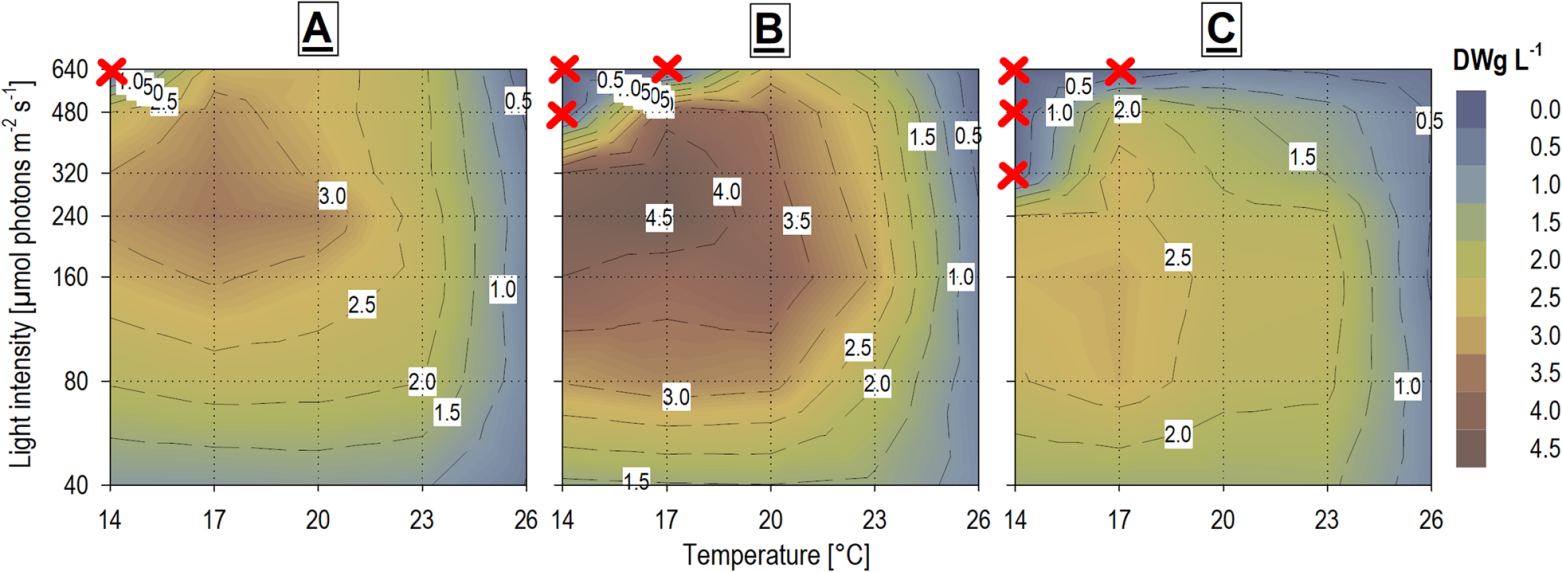
Heat-plot comparison of final harvested dry biomass yields (DWg L^−1^) of *H. magna* for the three temperature × light cross-gradient experiment replications: (**A**) RUN1; (**B**) RUN2; (**C**) RUN3. Red crosses indicate the conditions where the culture collapsed

The results of the cross-gradient experiment are presented as the means of three biological replications labeled RUN1, RUN2, and RUN3 with the variability between them also of importance. RUN1 provided average DW yields, but this replication differed from the other two in viability in border conditions when 34 out of 35 conditions (temperature × light combinations) exhibited survival and growth. Only the combination of the lowest temperature and highest light intensity was lethal (Fig. 1A). RUN2 performed overall highest DW yields and highest DW productivities for most of the conditions. The highest final DW yield (4.53 ± 0.05 g L^−1^) was obtained at 17 °C× 240 µmol m^−2^ s^−1^ (Fig. 1B) and the highest mean DW productivity (0.34 ± 0.00 g L^−1^ d^−1^) at 23 °C× 480 µmol m^−2^ s^−1^. On the other hand, at the low temperature and the high light intensities 3 cultures collapsed in this replication (Fig. 1B). RUN 3 was the worst in sense of overall DW yields, productivities, and culture viability. In the low temperatures and high light intensities 4 of the cultures failed to grow (Fig. 1C). The differences between individual replications were likely caused by the inoculum quality suggesting that optimization of inoculum preparation protocol is needed.

#### Growth rates, productivity, and final yields

**Fig. 2:**
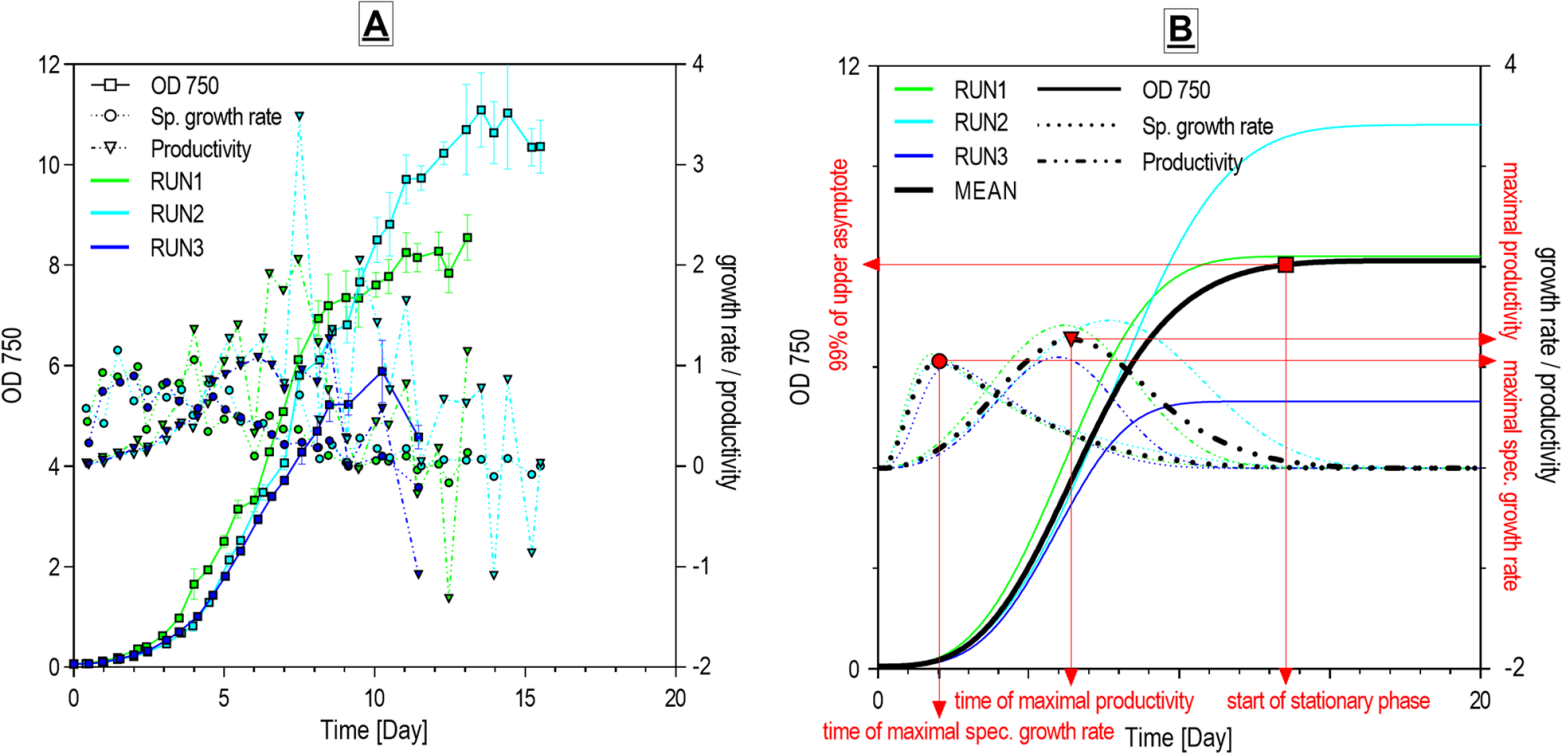
Example of the application of the Weibull growth curve equation to one of the cross-gradient conditions dataset (17 °C × 320 µmol m^−2^ s^−1^): (**A**) Values obtained from absorbance measurements (OD 750) and point-to-point calculated specific growth rate and productivity values from the raw data; (**B**) The same dataset after fitting the Weibull growth curve equation, with constructed specific growth rate and productivity curves, mean curves, and maximal values determination

**Fig. 3:**
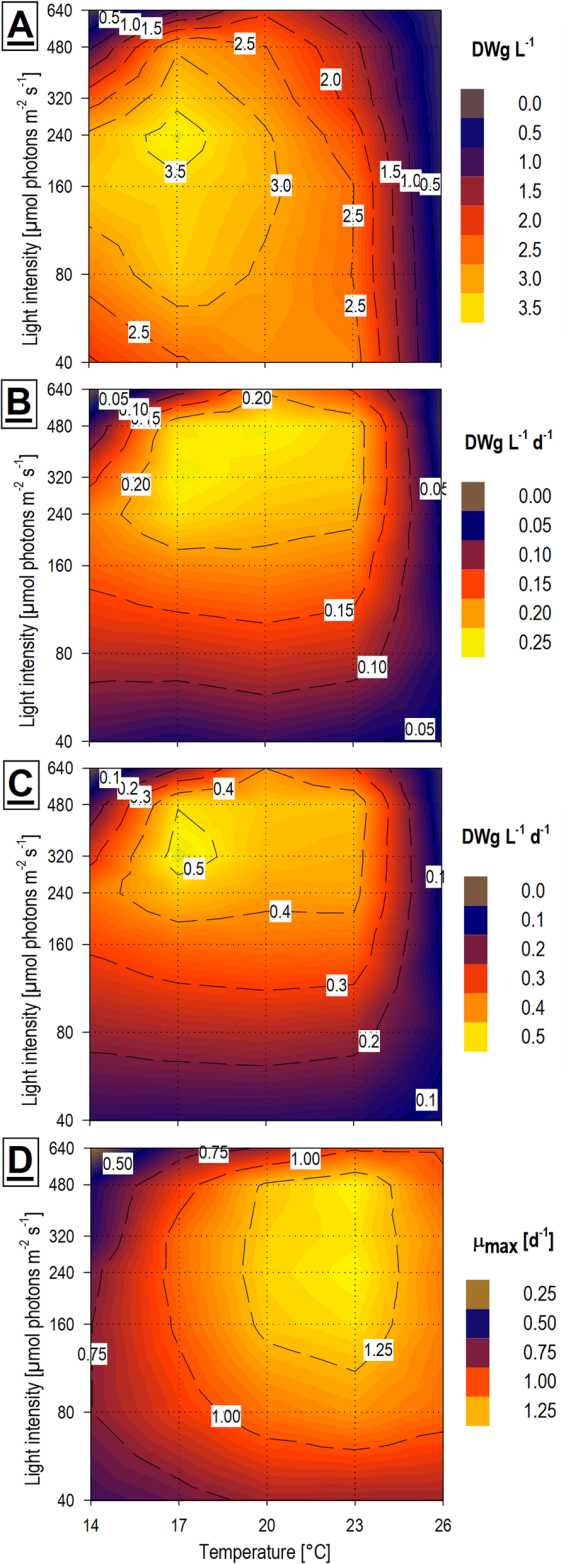
Growth and productivity of biomass of *H. magna* gained by Weibull growth curve equation utilization (n = 3) of the temperature × light cross-gradient visualized as heat-plots: **A** Expected final dry biomass yield (DWg L^−1^); (**B**) Mean dry biomass productivity (DWg L^−1^ Day^−1^); (**C**) Maximal dry biomass productivity (DWg L^−1^ Day^−1^); (**D**) Maximal specific growth rate (Day^−1^)

The growth parameters (final DW density, time of reaching the stationary phase, mean and maximal productivity, and maximal specific growth rate (Fig. 2)) were derived from the Weibull equation, which was based on the empiric OD 750 data and subsequently converted to DW values as described later (see Methods—Dry weight (DW) biomass calculation). The application of the model values appeared to be necessary due to the scattering between individual sampling points hampering proper calculation, especially in the case of productivity and specific growth rate maximal value determination. The expected final DW densities, as one of the major findings of this study, are depicted in a heat-plot (Fig. 3A). The optima for the highest final DW density were quite narrow and were localized at a temperature of 17 °C and medium light intensities from 160 to 320 µmol m^−2^ s^−1^. The highest expected final DW density was 3.73 g L^−1^ under conditions 17 °C × 240 µmol m^−2^ s^−1^. Densities over 3 g L^−1^ were obtained in a broader range of conditions from temperatures 14 to 20 °C and medium or lower light intensities between 160 to 240 µmol m^−2^ s^−1^. These values can be considered satisfying in comparison with other established FX and PUFA autotrophic producers (*Phaeodactylum tricornutum*, *Odontella aurita*) with reported densities ranging between 0.49 to 6.36 g L^−1^ [19].

The estimated time of reaching the stationary phase (growth time) was significantly influenced (P = 0.0002) by light intensities more than by temperatures (P = 0.0140). It varied from less than 7 days under conditions of higher temperatures and higher light intensities to 35–45 days for the lowest light intensities and temperatures from 14 to 23 °C (Additional file 1: Fig. S1). Under conditions providing the highest DW yields, the growth time was about 15 days. Due to the slightly earlier harvest time (technical reasons) of the cultures grown at the lowest light intensities (40 µmol m^−2^ s^−1^), the higher influence of Weibull equation extrapolation is introduced into these results.

The highest Prod_mean_ were at similar conditions as those for the highest final DW densities (Fig. 3B), but this optimum was also extended to the higher light intensities and the higher temperatures. The total highest Prod_mean_ was 0.25 g L^−1^ d^−1^ under conditions 17 °C  × 320 µmol m^−2^ s^−1^, and very similar Prod_mean_ values were under conditions 20 °C  × 480 µmol m^−2^ s^−1^ (Fig. 3B). The Prod_mean_ of the batch culture is highly influenced by the growing time. Cultures usually have low productivity at the beginning of growth when the culture is highly diluted and again at the end when limiting factors suppress the growth rate (Additional file 2). To prospective increase of the Prod_mean_, it is favorable to cut off the lag and stationary phases of the batch culture and take advantage of the middle growth phase when productivity is approaching the Prod_max_ values. The desired goal is to balance culture technique utilizing the continuous culture mode of the chemostat or turbidostat at the Prod_max_ values [33], however, determining the expected Prod_max_ values and factors influencing it is a prerequisite.

The Prod_max_ values for *H. magna* were also calculated based on the mean Weibull model growth curves (Fig. 2B). The highest Prod_max_ were observed at temperatures from 17 to 23 °C and light intensities from 240 to 480 µmol m^−2^ s^−1^. In this quite broad range of conditions, the Prod_max_ did not drop below the value of 0.41 g L^−1^ d^−1^ (Fig. 3C), and this optimum range had a clear peak at the value of 0.54 g L^−1^ d^−1^ under conditions 17 °C × 320 µmol m^−2^ s^−1^. However, these calculations are based on the average of all three replications thus they are negatively influenced by RUN3 which tended to fail to grow at high light intensities. Hence it is reasonable to expect that the Prod_max_ could be even greater at higher light intensities. If we consider only RUN1 and RUN2 for the calculations, the Prod_max_ was reaching values of 0.63 g L^−1^ d^−1^ under conditions 20 °C × 480 µmol m^−2^ s^−1^. The time when cultures achieved Prod_max_ was significantly influenced by temperature (P < 0.0001) but not by light intensity (P = 0.0651) and Prod_max_ values were negatively correlated to both conditions (Additional file 1: Fig. S1). This means that in lower temperatures and lower light intensities, the Prod_max_ was achieved later. At the temperatures of 14 °C, 17 °C, 20 °C, and 23 °C Prod_max_ were reached on average after 9 days, 7.5 days, 5.5 days, and 4.5 days, respectively. Most of the cultures did not considerably grow at 26 °C. Volumetric or areal biomass productivity is naturally one of the keystones of applied phycology [34]. In this parameter, *H. magna* cannot compete with the fastest-growing microalgae which can achieve volumetric productivities exceeding values of 1.29 (Prod_mean_) and 2.70 (Prod_max_) g L^−1^ d^−1^ for Chlorellaceae [35]. However, *H. magna* possesses significant added value due to high-value compound production, so the presented biomass productivity is solid in this context. The usual volumetric biomass productivity of FX-producing microalgae in the photoautotrophic batch mode cultivation is between 0.03 and 0.49 g L^−1^ d^−1^ [12].

A broad optimum of µ_max_ was found for the temperatures from 20 to 23 °C and for light intensity from 160 to 480 µmol m^−2^ s^−1^ with higher µmax values slightly inclined to the higher temperatures (Fig. 3D). The µ_max_ values exceeded 1.25 d^−1^, which corresponds to a doubling time of 13.3 h. Because the µ_max_ values were similar for more variable culture conditions and were not much influenced by light intensity, we can assume that these values could come close to the maximal physiologically achievable doubling time for *H. magna*. The highest specific growth rates lasted just for a short period (≈ 10 h) usually at the beginning of the experiment (Additional file 2). The time of reaching the µ_max_ was significantly correlated (P = 0.0001) with temperature increase and less strongly correlated (P = 0.0131) with a light decrease (Additional file 1: Fig. S1). For most conditions, no acclimatization phase was observed, and exponential growth started soon after the inoculation. However, a quite long (3–4 days) acclimatization phase was recorded for the cultures at lower temperatures and highest light intensities, and the exponential growth phase started significantly later. This phenomenon was the cause of the observed negative correlation between light intensity increase and the time of µ_max_. The acclimatization to these stress conditions seems to be difficult to overcome by *H. magna* cultures and in some experiment replications the growth was substantially postponed (RUN1), or the cultures collapsed in the others (RUN2 and RUN3).

To conclude, the temperature of 17 °C and higher light intensities (between 320 and 480 µmol m^−2^ s^−1^) represented the conditions giving the highest biomass yields and had the highest Prod_mean_ and Prod_max_. Hence, we can consider these culture conditions as the best for the biomass productivity of *H. magna* in our batch mode setup. At the higher temperature (20 °C) the Prod_mean_ and Prod_max_ reached similar values, but the growth time was shorter which caused lower biomass yields. It is important to note however that the final densities and productivities achieved in the presented study were associated with the precise culture system, so lower volumetric yields can be expected for a larger scale where such fine control cannot be provided [34]. For a detailed overview of the calculated curves see Additional file 2.

#### Cell size

**Fig. 4:**
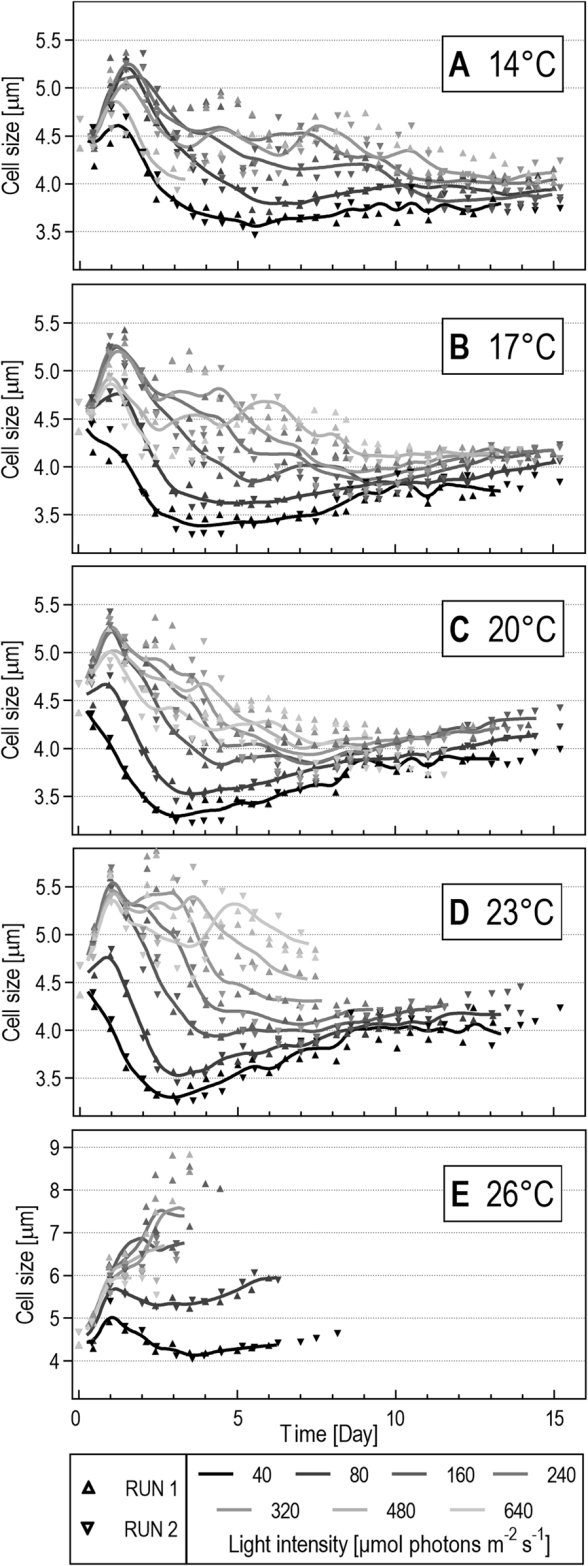
Cell size (μm) of *H. magna* at different culture conditions of the temperature × light cross-gradient. Only data from RUN1 and RUN2 replications were used for mean cell size calculations

In addition to OD 750, cell count and cell size were measured for the cross-gradient experiment. These parameters were subsequently used to calculate the culture biovolume and the Weibull equation was applied to construct the biovolume growth curve as well. However, these calculations provided similar growth curves as for OD 750 and thus they were not used for other applications, but were used separately to show a deeper insight into *H. magna* growth. For most of the cultures, a short acclimatization lag phase (about 24 to 48 h from the beginning of the experiment) was observed. During this initial phase cell division was limited but the cell sizes increased which led to a considerable increase in the culture biovolume as well as OD 750. After this acclimatization phase cell division started which led to a cell size decrease (Fig. 4). This decrease in size was not uniform for all conditions. Generally, cell size was influenced by light intensity; cells were larger at higher light intensities. This effect can be illustrated at 23 °C, where the most prominent differences were observed (Fig. 4D). The mean cell size at the light intensity of 40 µmol m^−2^ s^−1^ was less than 3.5 μm in diameter, but at the light intensity of 480 µmol m^−2^ s^−1^, it was almost 5.5 μm. After the initial drop in cell size during the acclimatization phase, cells slowly increased to about 4.0 μm in diameter, which appeared to be the typical cell size in older stationary cultures. Cell count usually stopped rising earlier than the OD 750 values, which can be attributed to cell size increase during the late growth phase.

The development of cell sizes at the highest temperature of 26 °C was different from the rest of the temperature variants. Cell division was limited, and cells increased to a much larger size (Fig. 4E). Only at the low light intensities, a minor cell number increase was observed. Observed phenomenon was probably caused by a stress reaction to the higher temperature. Thus, this temperature can be considered irrelevant to the production of *H. magna* biomass. Cell size variability ranging from 3.2 µm to 7.5 µm in diameter, and modulations connected to light, temperature, and culture age are indications of *H. magna* metabolic plasticity.

#### Fucoxanthin (FX)

**Fig. 5:**
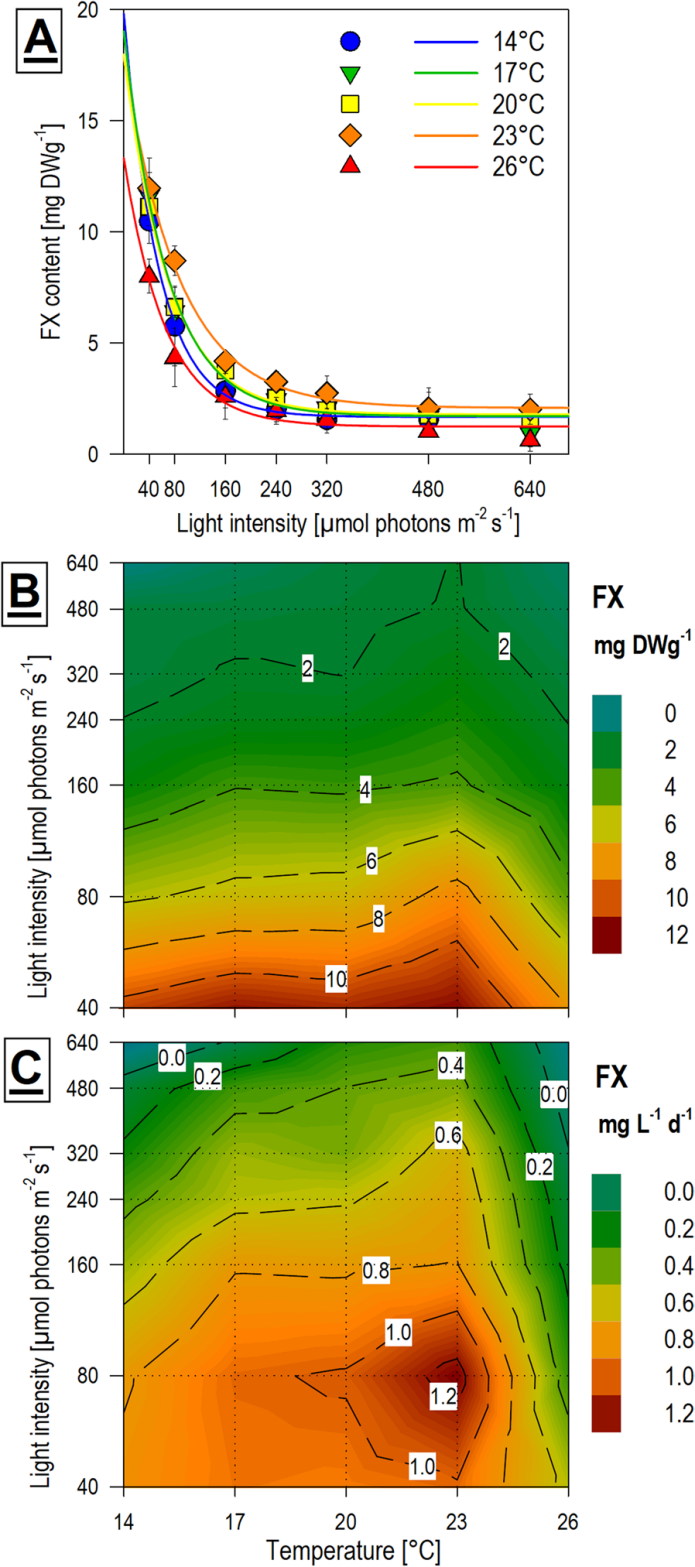
Mean (n = 3)* fucoxanthin (FX) content and volumetric productivity of *H. magna* obtained from final harvested biomass of the temperature × light cross-gradient: (**A**, **B**) total FX content (mg DW g^−1^); (C) FX volumetric productivity (mg L^−1^ d^−1^). *In conditions of low temperature and high light intensities, not all three replications were available (for detail see Fig. 1)

FX, the major target product of this study, was strongly affected by light intensity, while the effect of temperature was less prominent (Fig. 5A, B). These findings agreed with comparable research [7, 12]. FX content in microalgae is significantly modulated by photosynthetically active radiation due to FX being a component of the photosynthetic antenna and taking part in light-harvesting systems [36]. Algae generally react to low light conditions by increasing the quantity of photosynthetic pigments complexes [37] which can even be observed at the level of gene expression [38]. Unlike the results of biomass productivity and culture viability, the differences in FX content among the three experiment replication were minor. Thanks to the fine gradient of light intensities tested in this study we found an exponential increase of FX content with decreasing light intensity (Fig. 5A). This finding suggests that only a mild modulation of light intensity can impact *H. magna* FX productivity dramatically, especially at light intensities below 80 µmol m^−2^ s^−1^.

The highest mean FX content per final harvested biomass was recorded under the conditions of 23 °C × 40 µmol m^−2^ s^−1^ (Fig. 5B) reaching values of 11.98 ± 1.10 mg DWg^−1^. The overall highest FX content of 12.74 ± 2.57 mg DWg^−1^ was recorded in the experiment inoculum culture, which suggests that the total FX content in *H. magna* biomass can be higher than it was achieved in the cross-gradient experiment. Even though temperature did not show such a strong effect on the final FX content, cultures at 23 °C showed slightly higher content than the others in the tested gradient. Due to the slow growth of *H. magna* at the lowest light conditions, the optimum of mean FX productivity was shifted to slightly higher light intensities than the highest absolute FX content (Fig. 5C). There was quite a narrow peak of highest FX productivity under conditions of 23 °C × 80 µmol m^−2^ s^−1^. Mean FX productivity within this optimum was 1.27 mg L^−1^ d^−1^. If we consider the results of the growth curves where the Prod_max_ values were approximately double that of the Prod_mean_, we can expect that the maximal productivity of FX was probably double as well.

Achieved values of *H. magna* FX content and productivity were comparable with other more established microalgae producers such as *Phaeodactylum tricornutum*, *Odontella aurita*, and *Tisochrysis lutea*. Recent reviews focused on FX microalgae producers [4, 7, 12] reported that total FX content can vary significantly from less than 1 mg DWg^−1^ to a maximum of 79.4 mg DWg^−1^. Recorded productivities are similarly variable, ranging from 0.04 to 16.5 mg L^−1^ d^−1^. The highest achieved FX content and productivity reported for *Tisochrysis lutea* and *Nitzschia laevis* were associated with hetero-mixotrophic lab-scale and highly controlled and optimized cultivation systems [33, 39, 40]. The only comparable record for autotrophic freshwater chrysophyte, *Mallomonas kalinae*, evaluated an FX content of 26.6 mg DWg^−1^ during dim light conditions [25]. This result further pinpoints the potential of chrysophytes for FX production, although FX productivity was not determined for *M. kalinae*.

Interesting trends in FX content during *H. magna* cultivation were observed from periodical sampling every second day of growth. These data showed that at lower light intensities (40 and 80 µmol m^−2^ s^−1^) FX content per DW increased in time, while at higher light intensities FX content decreased in time. These trends were similar at all temperatures (Additional file 1: Fig. S2).

#### Fatty acids (FA)

Cultivation of algae for lipid production is a broadly studied topic, mainly thanks to the potential of microalgae-based-biofuel production [41], as well as due to the health benefits of individual FA and PUFA especially [13]. In past decades many authors, therefore, focused on the FA content in microalgae and lipid production concerning different temperatures, light intensities, and other factors [34]. However, comparative studies focused on the effect of culture conditions on FA composition exclusively across class Chrysophyceae are missing. There are however some general FA profiles from culture collection strains available also for chrysophytes [42, 43] and according to these findings, the *H. magna* FA profile is mostly consistent with other autotrophic chrysophytes. For a detailed overview of the *H. magna* FA profiles see Additional file 3.

**Fig. 6:**
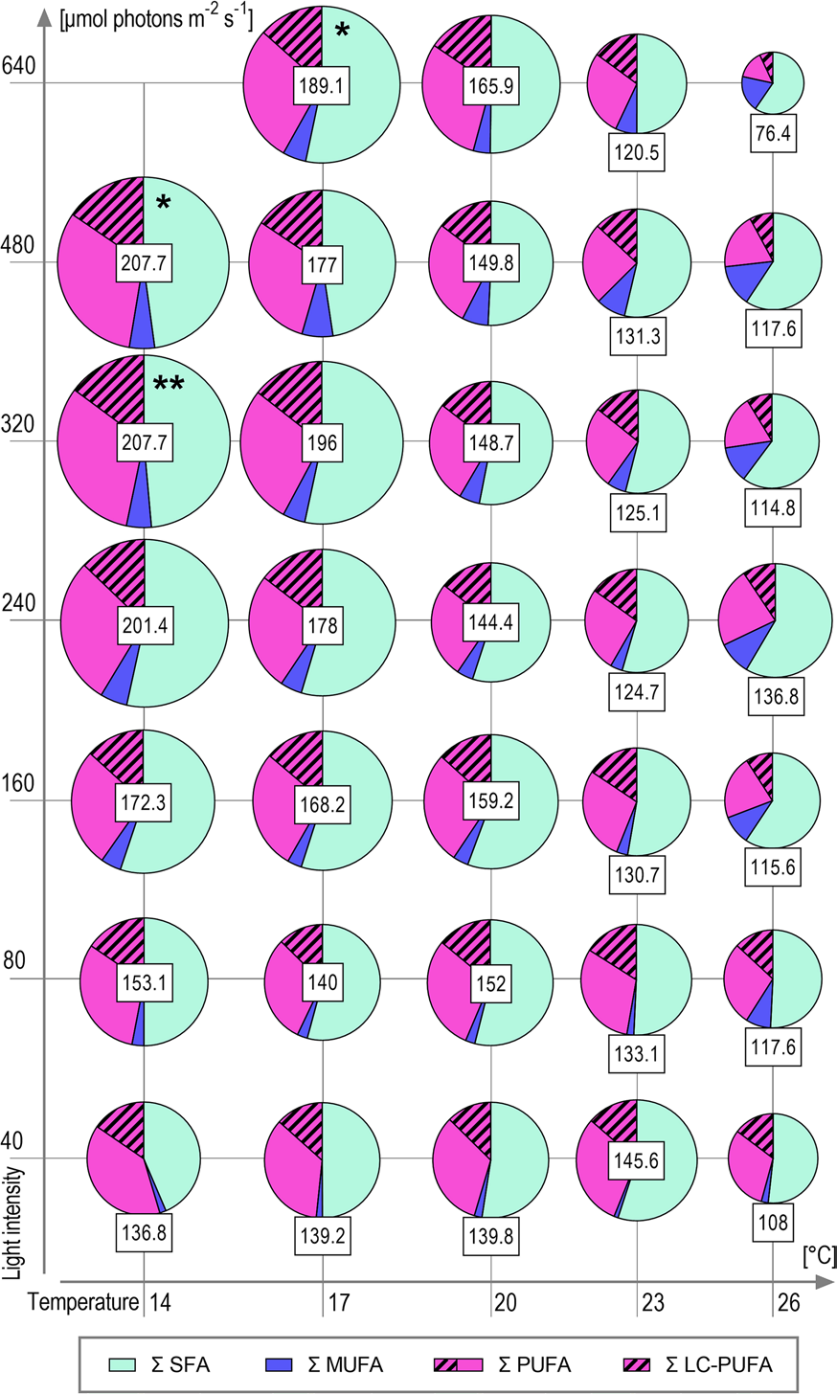
Quantity and proportions of major groups of fatty acids (FA) obtained from final harvested biomass of the temperature × light cross-gradient. Each pie plot represents the mean (n = 3) FA content of a single culture condition. The sizes of pie plots and their sections are proportional to the total FA amount and the number in boxes represents the quantity of total FA per biomass (mg DWg^−1^). Color coding corresponds to the major FA groups: saturated FA (SFA); monounsaturated FA (MUFA); polyunsaturated FA (PUFA); and long-chain PUFA (LC-PUFA) as a subgroup of PUFA. * One replication available (n = 1); ** Two replications available (n = 2)

We did not analyze the functional distribution of the FA and proportions among major lipid groups such as polar and neutral lipids because the overall productivities of FA and PUFA, rather than their biological function, were the focus of the presented study. The highest total FA content per final harvested biomass was at the lowest tested temperature (14 °C) and mid to high light intensities (240–480 µmol m^−2^ s^−1^). Under these conditions the average total content of FA slightly exceeded 200 mg DWg^−1^ (Fig. 6). However, it is needed to be mentioned that at high light and low temperature (conditions most favorable for FA accumulation) certain instability of the growth was observed as it is described above (see Results and discussion—Variability among experiment replications).

The optimal mean productivity of total FA was slightly shifted to higher temperatures compared to the maximal FA amount because the growth rate was suppressed at the lowest temperature. The optimum for total FA productivity was at 17 °C with a light intensity of 320 µmol m^−2^ s^−1^ which was the same optimum as applied for the overall biomass productivity (Fig. 3B, C). If we compare the maximal total FA amount achieved (~ 207.7 mg DWg^−1^) and maximal total FA mean productivity (~ 51.3 mg L^−1^ d^−1^) with other biotechnologically utilized microalgae, there is still a substantial gap between them and our values. For instance, Přibyl et al. [35] reported a maximal lipid content of 638 ± 41 mg DWg^−1^ and a maximal volumetric lipid productivity of 1 425 ± 135 mg L^−1^ d^−1^ for *Chlorella vulgaris* in laboratory conditions and lipid productivity of 326 ± 10 mg L^−1^ d^−1^ for the same strain in a 150 L volume thin layer cultivation unit in a greenhouse. Although *H. magna* had a lower productivity of lipids in comparison with most relevant lipid-producing microalgae, it produces other valuable compounds like FX simultaneously, and its FA profile, namely the amount and PUFA composition make this alga important. Further cultivation technology optimization may also enhance its FA and PUFA productivity.

Comparison of the FA according to saturation revealed that saturated FA (SFA), monounsaturated FA (MUFA), and PUFA did not show strong differences in proportions among each group concerning the cultivation conditions. The only exception was the prominent difference in the poorly growing cultures at the highest temperature (26 °C) and higher light intensities, where the increase of MUFA was accompanied by a decrease in PUFA (Fig. 6). As the proportion between each group of FA did not considerably differ, the same production optima for total FA (17 °C × 320 µmol m^−2^ s^−1^) also applies to each FA group production optima. *H. magna* reached satisfyingly high contents and mean productivities of PUFA reaching the maximal value of ~ 98.4 mg DWg^−1^ and ~ 21.5 mg L^−1^ d^−1^ respectively. For comparison, Cepák et al. [44] reported the highest autotrophic EPA productivities of ~ 30 mgL^−1^ d^−1^ for oleaginous freshwater eustigmatophycean (Stramenopila) microalgae *Trachydiscus minutus*, which is a strain known for its considerably high EPA content as its dominant PUFA.

**Fig. 7:**
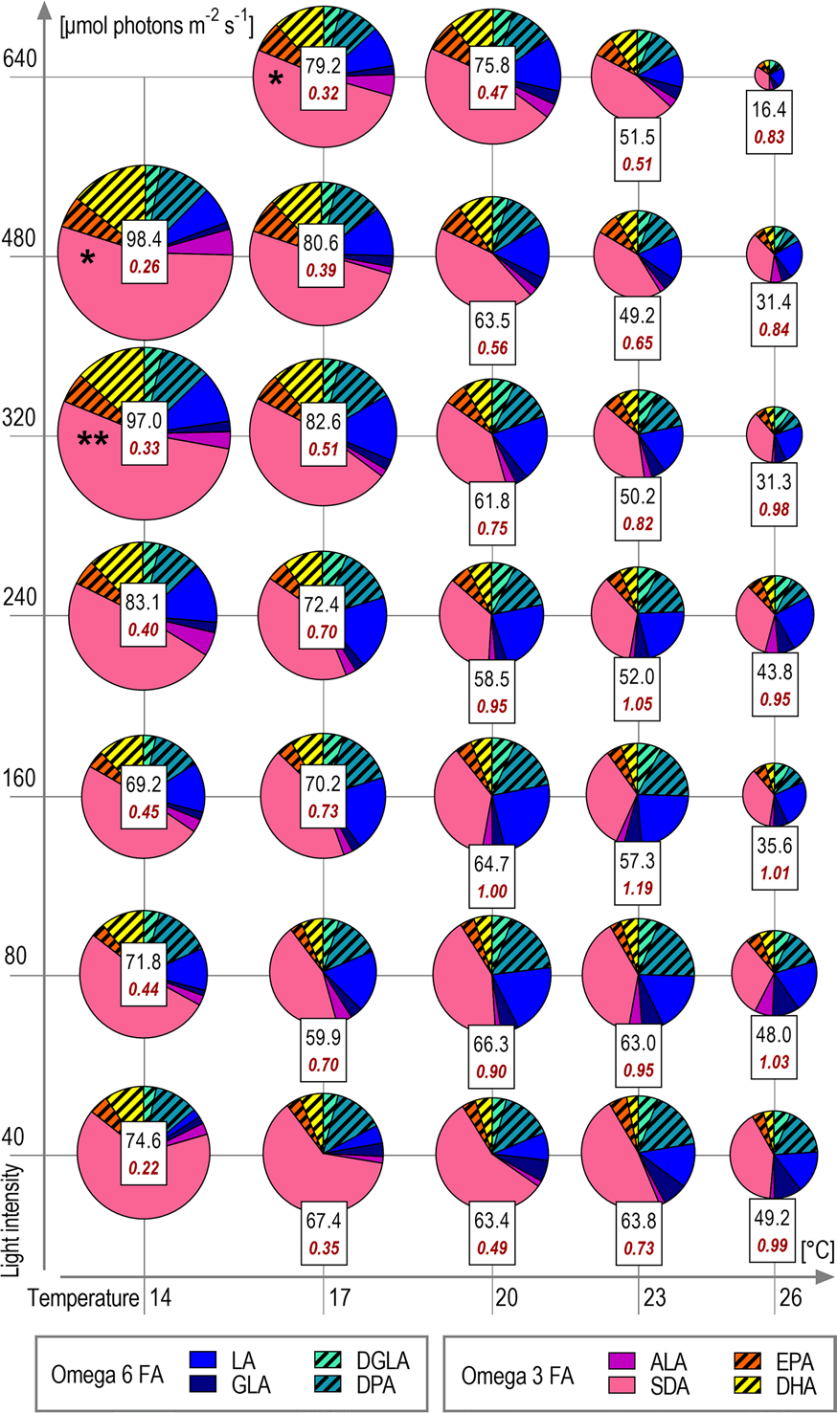
Quantity and proportion of individual Omega3 and Omega6 polyunsaturated fatty acids (PUFA) obtained from final harvested biomass of the temperature × light cross-gradient. Each pie plot represents the mean (n = 3) PUFA content of a single culture condition. The sizes of pie plots and their sections are proportional to the amount of FA. The black number in the box represents the quantity of total PUFA content per biomass (mg DWg^−1^) and the red bold number represents the Omega6: Omega3 PUFA ratio. Colors correspond to the individual PUFA—blue/green colors indicate Omega6 PUFA and red/yellow colors indicate Omega3 PUFA. Long-chain PUFA (LC-PUFA) are marked by diagonal hatching. PUFA of occurrence are Linoleic acid—18:2ω6 (LA), Gamma-linolenic acid—18:3ω6 (GLA), Dihomo-gamma-linolenic acid—20:3ω6 (DGLA), Docosapentaenoic acid—22:5ω6 (DPA), Alpha-linolenic acid—18:3ω3 (ALA), Stearidonic acid—18:4ω3 (SDA); Eicosapentaenoic acid—20:5ω3 (EPA); Docosahexaenoic acid—22:6ω3 (DHA). * One replication available (n = 1); ** Two replications available (n = 2)

The PUFA profile of *H. magna* was remarkably diverse consisting of four Omega 3 PUFA (ALA 18:3ω3, SDA 18:4ω3, EPA 20:5ω3, and DHA 22:6ω3) and four Omega 6 (LA 18:2ω6, GLA 18:3ω6, DGLA 20:3ω6, and DPA C22:5ω6) in a non-negligible amount (more than 1.0 mg DWg^−1^). All mentioned PUFA are essential or semi-essential compounds involved in mammalian FA metabolism [16]. While the content of Omega 3 PUFA showed similar characteristics as applied for the total FA content and their maximum was 78.1 mg DWg^−1^ in conditions 14 °C × 480 µmol m^−2^ s^−1^, the situation in Omega 6 PUFA was different. Omega 6 PUFA had a broader optimum placed in higher temperatures and lower light intensities with maximal content of 32.3 ± 6.2 mg DWg^−1^ under the conditions 20 °C × 160 µmol m^−2^ s^−1^. These differences in the pattern of Omega 3 and Omega 6 PUFA content resulted in the considerable variability of the Omega 6 to Omega 3 PUFA ratio which ranged from 0.22 under the lowest light intensities and lowest temperature (14 °C × 40 µmol m^−2^ s^−1^) to 1.19 in conditions 23 °C × 160 µmol m^−2^ s^−1^ (Fig. 7).

**Fig. 8:**
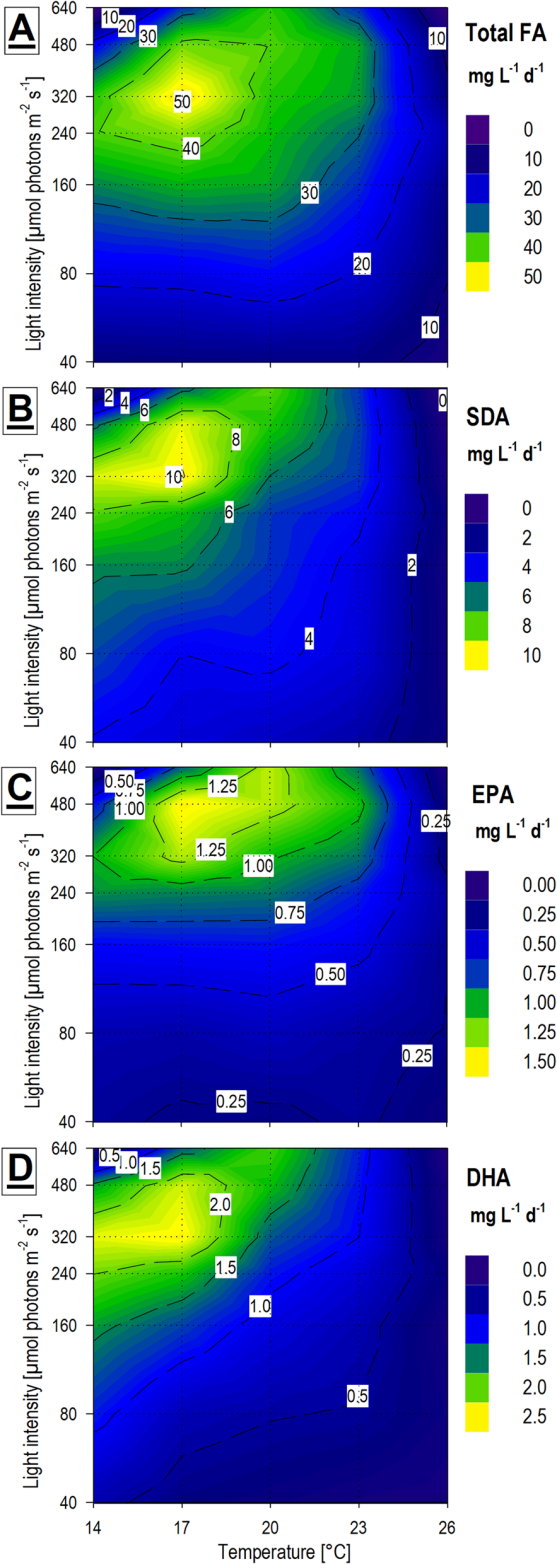
Mean (n = 3)* volumetric productivities of fatty acids (FA) obtained from final harvested biomass samples of the temperature × light cross-gradient visualized as heat-plot: (**A**) Total FA productivity (mg L^−1^ Day^−1^); (**B**) Stearidonic acid—18:4ω3 (SDA) productivity (mg L^−1^ Day^−1^); (**C**) Eicosapentaenoic acid—20:5ω3 (EPA) productivity (mg L^−1^ Day^−1^); (**D**) Docosahexaenoic acid—22:6ω3 (DHA) productivity (mg L^−1^ Day^−1^). *In conditions of low temperature and high light intensities, not all three replications were available (for detail see Fig. 1)

The PUFA of main interest were those with the highest economic value (EPA and DHA) and the most abundant (SDA). EPA content presents a slightly different pattern than the rest of the Omega3 PUFA. It was accumulated not just at low temperatures and high light but also at higher temperatures with a maximal content of 6.3 ± 1.1 mg DWg^−1^ under conditions of 17 °C × 480 µmol m^−2^ s^−1^. DHA total content was usually approximately two times higher than the EPA (Fig. 7) with a similar accumulation pattern as the total FA. Maximal DHA content was 14.1 mg DWg^−1^ (n = 1) under conditions 14 °C × 480 µmol m^−2^ s^−1^. Productivities of both PUFA were shifted towards higher temperatures due to the slow growth at low temperature, being ~ 1.5 mg L^−1^ d^−1^ under conditions of 17 °C× 480 µmol m^−2^ s^−1^ for EPA (Fig. 8C) and ~ 2.5 mg L^−1^ d^−1^ under conditions of 17 °C× 320 µmol m^−2^ s^−1^ for DHA (Fig. 8D). SDA represented about 72% of all Omega3 PUFA content on average. The highest content of SDA was recorded in two separate optima, i.e. 14 °C× 480 µmol m^−2^ s^−1^ and 14 °C × 40 µmol m^−2^ s^−1^ yielding 53.6 mg DWg^−1^ (n = 1) and 48.6 ± 2.7 mg DWg^−1^ under these conditions, respectively. The optimum for SDA productivity was again shifted towards the higher temperatures and was ~ 10.1 mg L^−1^ d^−1^ under conditions of 17 °C × 320 µmol m^−2^ s^−1^ (Fig. 8B).

Most of the previous research focused on the combined production of FX and PUFA described FX and EPA production in diatoms [45, 46] or FX and DHA in haptophyta [47], these PUFA being dominant in respective taxa. Although EPA and DHA content was lower in *H. magna*, dominant SDA is one of the important Omega 3 PUFA as well, being required for the human organism [48, 49] with the potential for medical use [50]. Furthermore, currently there is no information about SDA industrial production potentiality by photoautotrophic microalgae. It is possible that microalgae-based SDA production can be more suitable than production by land plants, where the SDA content per biomass cannot compete with focused microalgae production. *H. magna* could be a promising candidate for algae-based SDA production.

### Prospects

The productivities of individual high-value compounds make *H. magna* and related chrysophyte algae interesting candidates for industrial development. The simultaneous manufacture of several high-value products is certainly one of the viable strategies to valorize the produced biomass and reduce the cost of individual components. The concept of utilizing microalgae biomass as a crude material for subsequent co-extraction of multiple high-value compounds has been recently under discussion [19, 51]. With this respect, it is worth mentioning that Chrysophyceae are producers of other interesting compounds such as chrysolaminarin and extracellular polysaccharides and thus their production can be expected in *H. magna.* Our preliminary assessment shows that indeed *H. magna* intensely produces extracellular polysaccharides (Additional file 1: Fig. S3). A separate study dedicated to this topic would certainly confirm the further biotechnological potential of this alga to co-produce several high-value products.

However, the results of the cross-gradient experiment demonstrate that there was not a single optimum for the concurrent maximal production of FX and PUFA. The highest accumulation of FX was at dim light and higher temperatures and the accumulation of PUFA was at higher light intensities and lower temperatures. Which conditions are more advantageous to choose depends on the many variables such as downstream processing methods, the economic value of the target product, energy supply, geographic localization, and overall design of the cultivation technology. Generally, it could be expected that higher temperatures and lower light intensity are less energy demanding and so the cultivation conditions preferable for FX production could be more profitable. Despite FX being in lower concentrations in biomass than PUFA, it has a greater economic value. Furthermore, even under the conditions optimal for FX productivity, there were still reasonable amounts of PUFA, which does not apply in the opposite situation. One of the options could be also the application of two-stage cultivation connecting the production optima for FX and PUFA. However, based on our preliminary experiments, *H. magna* does not cope well with a sudden change in temperature/light conditions (Additional file 1: Table S1, Fig. S4) and the resulting productivities were lower compared to batch cultures maintained at constant conditions.

When considering FX as a single high-value component of *H. magna* biomass the following strategies can be considered for the further enhancement of FX productivity: (1) Employing a continuous or semi-batch cultivation mode to utilize the peak of maximal productivity; (2) Media optimization because the sufficiency of nitrogen plays an important role in FX accumulation in microalgae [9]; (3) Adjusting the photoperiod [37] or light spectra [52]; (4) Utilizing mixotrophic growth as the tested strain should be able to take an organic substrate, according to its phylogenetic position [53]. From above mentioned we have experimentally proved that the light spectrum effects on *H. magna* FX content, with blue light providing the highest FX content per DW, and purple light providing the highest FX volumetric productivity (Additional file 1: Fig. S5), and thus this aspect should also be considered when designing production technology.

*H. magna* has also the potential as a model organism for other experimental research of freshwater autotrophic chrysophytes as not many species of this group have been studied yet. It showed a good ability to grow in laboratory conditions, where it performed predictably and usually showed stable growth if treated carefully. On the other hand, it proved to be less robust to cultivation conditions instability and we were not able to maintain it on the slant agar. *H. magna* is tolerant to quite broad culture conditions and can modulate its metabolism and acclimate to specific culture conditions which facilitates further research of the physiology of chrysophyte algae. Chrysophyceae are generally an understudied taxon of photoautotrophic eukaryotes even though they are very common members of freshwater communities, namely those of lower pH, and they are an important part of the spring phytoplankton communities [54].

## Conclusions

The present study shows detailed pioneer research on FX and PUFA production in chrysophyceaen algae using *Hibberdia magna*. We have characterized optima for biomass and our target compound production using an extensive batch mode cultivation experiment on a cross-gradient of temperature and light. We reveal that *H. magna* maximal biomass productivities are favorable for large-scale cultivation due to presence of multiple high-value components. Our study shows that freshwater photoautotrophic chrysophytes should be considered for FX and PUFA production technology in inland areas where the preparation of sea-water-based culture media is not optimal. Finally, we propose that *H. magna* can serve as a model strain for further physiological and biotechnological studies in photoautotrophic Chrysophyceae as no such organism is established.

## Methods

### Algal strains screening

The studied alga *H. magna* was selected based on a broader screening of available strains from public algal culture collections. Initially, we prepared a list of freshwater Ochrophyta and Haptophyta flagellate strains accessible from the main world algal culture collections (SAG—Germany; CCAP—UK; NORCCA—Norway; CCAC—Germany; UTEX—USA; NCMA—USA; NIES—Japan; CCCM—Canada; RCC—France; CCALA—Czechia). This list contains over a hundred species belonging to more than fifty genera, mostly from the class Chrysophyceae but also a few members of the classes Raphidophyceae (Stramenopila), Pavlovophyceae (Haptophyta) as well as some *incertae sedis* members. Strains were further selected according to parameters that predetermined the organisms as potentially exploitable for cultivation. The parameters were the following: (1) Photoautotrophic; (2) Single-cell (non-filamentous or colonial) with small cell dimensions; (3) Not forming extracellular structures such as polysaccharide sheets, lorica, scales, or spines; (4) Not originating from extreme or polar habitats; (5) Maintained in more culture collections. According to these parameters, 10 strains were selected and ordered from 4 culture collections (Table 1). The strains were then subjected to pilot growth tests: (1) growth in 100 mL Erlenmeyer flasks in a culture chamber at 18 °C; without shaking; supplied with a constant dim light of ~ 20 µmol photons m^−2^ s^−1^ (µmol m^−2^ s^−1^); (2) growth in 170 mL culture tubes at 21 °C; mixed by bubbling with air supplemented with 1% CO_2_; with a constant moderate light of ~ 200 µmol m^−2^ s^−1^ in the culturing system described further (see Methods-Experimental design). Cultivation media used for the pilot growth tests were the same as indicated for maintenance in the respective culture collection. The resulting biomasses were analyzed for the target products (FX, PUFA, LC-PUFA) to select the best candidate for further experiments.

### Selected strain, culture maintenance, and medium

The non-axenic algal strain of alga *Hibbedia magna* K-1175 was obtained from the Norwegian Culture Collection of Algae (NORCCA). The culture was maintained in 100 mL Erlenmeyer flasks in a liquid medium without shaking at 18 °C and a constant dim light intensity of ~ 20 µmol m^−2^ s^−1^. It was necessary to re-inoculate the culture into a fresh medium regularly (approximately every 2 months) as old cultures tended to collapse. The culture medium for preservation and inoculum preparation differed from that used for final experiments (Additional file 1: Table S2) but all media were modifications of the original WC medium [55], which was selected based on initial growth tests. WC medium of all used modifications was as clear as distilled water with no precipitants being observed. The inoculum for experiments was prepared in a 10 L bottle (providing more stable growth compared to Erlenmeyer’s flasks) filled with 6 L of culture and cultivated for 4–5 weeks before the experiment, at 18 °C at the constant dim light intensity of ~ 40 µmol m^−2^ s^−1^ provided by a white fluorescent lamp at one side of the bottle. The culture was mixed by bubbling with air sterilized by filtration (Filter Sartorius Midisart 2000, 0.2 µm PTFE, Typ: 17805) and enriched by CO_2_ to the concentration of 1% (v/v).

### Experimental design

A cross-gradient of temperature and light [56] was performed to evaluate the effect of these major environmental factors on growth and high-value compound productivities. The cultivation experiment was performed in three biological replications. The individual replications followed each other in time. According to the pilot growth tests, five different temperatures and seven different light intensities were selected, accounting for 35 different culture conditions. The temperature was regulated by a water-bath-controlled system set to 14 °C, 17 °C, 20 °C, 23 °C, and 26 °C. Due to certain hysteresis of the temperature controlling system, the temperature varied ± 0.5–0.75 °C. Every cultivation tube was illuminated by a separate vertical dimmable LED strip with a full white light spectrum. Light intensities were set based on measurements performed by the photometer (LI-250, LI-COR Environmental) equipped with a spherical probe placed in the middle of the cultivation tube filled with distilled water. All lights were set to the intensity of 40 µmol m^−2^ s^−1^ at the beginning of the experiment. Subsequently, the light was gradually increased to the final light intensities of 40, 80, 160, 240, 320, 480, and 640 µmol photons m^−2^ s^−1^, during the first 12 h. This treatment was performed to acclimatize the inoculum cultures to higher light conditions.

Cultivation was carried out in batch mode. The starting culture (cell density of approx. 1 mil. cells L^−1^ corresponding to the optical density at 750 nm (OD 750) ~ 0,06 was placed into round bottom cultivation tubes (170 ml culture volume, 40 mm diameter). The cultures were mixed by bubbling with filter-sterilized air (Filter Sartorius Midisart 2000, 0.2 µm PTFE, Type: 17,805) and enriched by CO_2_ to the concentration of 1% (v/v). The evaporation of media was compensated on demand by the addition of sterile distilled water. Approximately every 12 h a sample for OD 750 and cell count determination was collected from each culture. Moreover, every second day a sample containing exactly 0.5 mL of the culture suspension was collected for evaluation of FX content changes over time. OD 750 was monitored continuously. When the culture reached an early stationary phase (OD 750 values did not increase in a few consecutive measurements), the whole culture was harvested and the final DW per volume was determined. Cultivation time thus differs according to culture conditions.

### Biomass analyses

#### Sampling

Two types of samples were collected: (A) regular samples containing only minimal necessary volume of the culture were taken from culturing tubes using a syringe and a medical needle permanently placed in the culture vessel to prevent contamination by opening the tube; (B) final harvest biomass. Harvesting was done by the following steps: (1) bulk algae suspension samples were centrifuged in glass containers (1 670 g, 12 min, centrifuge Universal 320, Hettich); (2) The supernatant was discarded and the pellet was re-suspended in distilled water, and transferred into a 2 mL Eppendorf microtube and centrifuged again (12 100 g, 3 min, Mini spin centrifuge, model 5452, Eppendorf SE); (3) the supernatant was discarded and the resulting biomass was frozen at − 75 °C and lyophilized (Scanvac, CoolSafe); (4) Dried samples were kept at − 75 °C until subsequent FX and FA content analyses.

#### Culture density quantifications

For OD 750 determination, the culture samples were placed immediately after collection into 96-well plates (Nunc) in technical triplicates and the optical density was measured using a plate reader (FLUOstar Omega Plate Reader, BMG LabTech) at 750 nm. Distilled water was used as a blank as well as the diluting medium. If OD 750 exceeded the value of 0.9, the dilution of the sample was performed in the following (sample:water) ratios: 1:0, 1:3, 1:11, and 1:15 to get between OD 750 of 0.1 to 0.9.

For the DW density quantification, a known volume (between 2 and 10 mL—according to algal culture density) of well-mixed fresh algal culture was filtered through a pre-dried and pre-weighed glass microfibers filter (VWRI516-0870, 693, pore size 1.2 µm, 55 mm diameter, VWR international) using a vacuum filtration assembly. The filter was immediately dried in the oven at 90 °C for 8 h, kept in a desiccator, and weighed after cooling.

For the cell number determination, an aliquot of algal suspension sample was fixed by glutaraldehyde to a final concentration of 2.5%. Then, cell count and cell size were measured using a Multisizer4 Coulter Counter (Beckman Coulter, Inc.) and the resulting histogram-like data was semi-automatically evaluated using a custom Python script employing the Findpeaks package (Version 2.3.1). The most frequent cell size (modal diameter) was determined, and the cell count was quantified from the first convex point of the histogram to the final measured cell size of 20 μm. Biovolume (μL) was calculated from the mode cell diameter (volume of a sphere) multiplied by cell count.

#### Fucoxanthin (FX)

A known amount of freeze-dried biomass (3–4 mg) was weighed into 2 mL screw cap microtubes using an analytical balance (R160 P, Sartorius). Then roughly the same amount (volumetric) of 0.1 mm glass beads (Cat. No. 11079101, BioSpec Products) and 1.8 mL of ethanol were added into the microtube. Samples were homogenized using a Mini-Beadbeater-16 (Cat. No. 607EUR, BioSpec Products) by applying two 45 s cycles and centrifuged (12,100 g, 3 min, Mini spin centrifuge, model 5452, Eppendorf SE). Finally, 1 mL of centrifuged extract was transferred into screw cap glass HPLC vials. The extracts were immediately analyzed using a high-performance liquid chromatography (HPLC) system (Dionex UltiMate 3000 HPLC, Thermo Scientific, Carlsbad, CA, USA) equipped with an autosampler, column oven and diode array detector (DAD). Chromatographic separation was performed using a reversed-phase column (Luna^®^ C8 column, 100 × 4.6 mm, 3 μm, 100 Å) at 30 °C. The mobile phase consisted of a mixture of water (A) and methanol (B) which was pumped at a flow rate of 0.8 mL min^−1^ using a gradient elution as follows: 0–20 min, 20–0% A; 20–25 min, 0% A; 25–27 min, 0–20% A; 27–30 min, 20–20% A [57]. HPLC analysis was monitored at 450 nm. A commercial standard of FX (Sigma Aldrich, Darmstadt, Germany) was used for quantification and confirmation purposes.

The extraction and quantification procedure for FX content analysis of the continuous samples of the cross-gradient experiment was slightly different from the final harvested biomass samples. The whole sample of culture suspension of known volume (0.5 mL) and known OD 750 value was used for extraction. These samples were freeze-dried and extracted in 0.4 mL absolute Ethanol. The beadbeater procedure was replaced by 30 min of sonication (38 kHz) in an ice-cooled water bath sonicator (KrainTek, K-6LM). Then the samples were centrifuged (1 520 g, 5 min, centrifuge Universal 320, Hettich) and transferred onto a glass HPLC vial with an insert and the HPLC analysis followed as described above. Quantification of FX per DW was calculated according to the OD 750 to DW conversion coefficient (see Methods—Dry weight (DW) biomass calculation).

#### Fatty acids (FA)

A known amount of freeze-dried algal biomass (3–5 mg) weighed using an analytical balance (R160 P, Sartorius) was transferred into a screw cap glass test tube, 50 μg glycerol-tripentadecanoate was added as internal standard (ISTD) together with 1 mL 3 M HCl-methanol (w/w) and 2 mL methanol. Samples were homogenized in an ice-cooled sonicator (KrainTek, K-6LM) for 10 min. Transesterification was achieved by heating samples at 90 °C for 90 min and let cool at room temperature. After cooling 2 mL hexane was added, vortexed and sonicated for 10 min. Then 2 mL ice-cold 1 M NaCl was added, and samples were vortexed and centrifuged at 900 g for 10 min at 4 °C. The upper hexane phase was carefully transferred into a crimp-top vial. Quantitative and qualitative analysis of the FA was performed employing a gas chromatograph (Trace 1300, Thermo) equipped with a flame ionization detector (FID) equipped with HTA3000A autosampler (HTA, Italy). A TR-FAME column (60 m × 0.32 mm, df 0.25 μm) was used for separation with helium as a carrier gas, at a constant flow of 2 mL min^−1^. The temperature ramp was the following: starting temperature 140 °C; increased to 240 °C at 4.5 °C min^−1^ and then maintained at 240 °C for 10 min. The injector was kept at 260 °C and the detector at 250 °C. The retention times of FA methyl esters were compared to known standards (Supelco^®^ 37 Component FAME Mix; PUFA No.3 Supelco from menhaden oil), supplemented by the analytical standard of Stearidonic acid and all-cis-7,10,13,16,19 Docosapentaenoic acid methyl ester (both supplied from Cayman Chemical) which were not part of the original FAME mix. The amount of individual FA was calculated using ISTD.

### Data analysis and calculations

#### Growth curve characterization

The Weibull growth curve which is an exponential growth model with added inflection parameter [58] was fitted to the measured OD 750 data. This Eq. (1) is expressed:

1 $$D_{t} = D_{\infty } {-}(D_{\infty } {-}D_{0} )\exp \,({-}(kt)^{\delta } )$$

where *D*_*t*_ is culture density in time *t*; *t* is the time from the beginning of the experiment; *D*_*∞*_ is the upper asymptote; *D*_*0*_ is the lower asymptote; *k* is the growth rate; *δ* is a parameter that controls the inflection point.

The Weibull growth curve equation was used because (a) it is usually used for biological data [59]; (b) it allows smoothing the scattering between individual time points values and facilitates the subsequent calculation of other parameters; (c) this approach allowed us to average growth characteristics of the individual replications; (d) it fitted well with our data (Fig. 2); (e) this equation, unlike other growth curves equations, has the possibility to fix the starting point of the growth curve by specifying the lower asymptote value.

For the fitting of the growth curves, a two-step process was applied. The first step was performed using the software Growth II (Pisces Conservation Ltd.), where Weibull equation parameters were calculated. Subsequently, these parameters were transferred into Excel (Microsoft Corp.) where the lower asymptote value has been fixed to the measured starting OD 750 value and the final growth curve parameters and X–Y coordinates of the growth curve were calculated using the function SOLVER. The X–Y coordinates of the growth curve were determined on a 1 h scale basis (X-axis) and the curve was extrapolated beyond the time of the last measured data point. The Weibull growth curve was calculated for every three biological replications (cross-gradient experiment) and the resulting curves were averaged to the final mean growth curve.

Expected density when the selected culture reached the stationary phase of growth (expected final yield) was defined according to the constructed Weibull growth curves as a value equal to 99% of the upper asymptote. Subsequently, the time of stationary phase achievement (growth time) was defined as the time of reaching the value equal to 99% of the upper asymptote (Fig. 2B).

#### Productivity and specific growth rate

The volumetric productivity and specific growth rate values were calculated using values obtained from the average Weibull growth curve fitted to OD 750 data. This was used to define expected mean productivity (Prod_mean_) and maximal productivity (Prod_max_) more precisely.

The productivity (r_t_; Density L^−1^ d^−1^) was calculated for 1 h interval values based on the Weibull growth curve X–Y coordinates using Eq. (2):

2 $$r_{t} = {{\left( {D_{t} {-}D_{t - 1} } \right)} \mathord{\left/ {\vphantom {{\left( {D_{t} {-}D_{t - 1} } \right)} {({1 \mathord{\left/ {\vphantom {1 {24)}}} \right. \kern-0pt} {24)}}}}} \right. \kern-0pt} {({1 \mathord{\left/ {\vphantom {1 {24)}}} \right. \kern-0pt} {24)}}}}$$

where *D*_*t*_ is the Weibull growth curve density value at a given time (hour) and *D*_*t-1*_ is the growth curve density value at a given time -1 (hour). Subsequently, the productivity values enabled the construction of a productivity curve (Fig. 2B). Prod_max_ (Density L^−1^ d^−1^) was defined as the highest productivity value calculated for a 1 h interval for mean Weibull growth curve (Fig. 2B). Prod_mean_ (Density L^−1^ d^−1^) was calculated using Eq. (3):

3 $${\text{Prod}}_{{{\text{mean}}}} = {{(D_{99\% } {-}D_{0} )} \mathord{\left/ {\vphantom {{(D_{99\% } \_D_{0} )} {t_{99\% } }}} \right. \kern-0pt} {t_{99\% } }}$$

where *D*_*99%*_ is the density value of the Weibull growth curve at *t*_*99%*_; *D*_*0*_ is the density value at the beginning of the experiment; *t*_*99%*_ is the time of reaching the density value equal to 99% of the upper asymptote. Productivity of the target products (Prod_C_) (mg L^−1^ d^−1^) was not calculated from the Weibull growth curve *D*_*99%*_ values, but from the final harvested DW densities values using Eq. (4):

4 $${\text{Prod}}_{{\text{C}}} = {{(DW_{t} \,C_{t} {-}DW_{0} \,C_{0} )} \mathord{\left/ {\vphantom {{(DW_{t} \,C_{t} \_DW_{O} \,C_{O} )} t}} \right. \kern-0pt} t}$$

where *DW*_*t*_ and *DW*_*0*_ are dry biomass density at harvest and at the beginning of the experiment, respectively; *C*_*t*_ and *C*_*0*_ are the content of the target product per DW at harvest and the beginning of the experiment, respectively; *t* is the time of culture growth. For calculations of the mean productivities firstly the mean *DW*, *C,* and *t* were calculated from the triplicates, and subsequently the mean productivity of the target product was determined.

The specific growth rate (μ d^−1^) was calculated similarly to productivity for 1 h intervals based on the Weibull model curve X–Y coordinates using Eq. (5):

5 $${{\mu = }}{{{\text{ln(}}{{{\text{D}}_{t} } \mathord{\left/ {\vphantom {{{\text{D}}_{t} } {D_{t1} }}} \right. \kern-0pt} {D_{t{-}1} }})} \mathord{\left/ {\vphantom {{{\text{ln(}}{{{\text{D}}_{t} } \mathord{\left/ {\vphantom {{{\text{D}}_{t} } {D_{t\_1} }}} \right. \kern-0pt} {D_{t\_1} }})} {\left( {{1 \mathord{\left/ {\vphantom {1 {24}}} \right. \kern-0pt} {24}}} \right)}}} \right. \kern-0pt} {\left( {{1 \mathord{\left/ {\vphantom {1 {24}}} \right. \kern-0pt} {24}}} \right)}}$$

where *D*_*t*_ is the Weibull growth curve density value at a given time (hour) and *D*_*t-1*_ is the growth curve density value at a given time -1 (hour). The maximal μ value (μ_max_) (d^−1^) was defined as the highest μ value calculated for a 1 h interval for the mean Weibull growth curve (Fig. 2B).

#### Dry weight (DW) biomass calculation

Characterization of the culture growth was based on the OD 750 measurements as described above, but for better intelligibility of the results these data were converted to the DW (g L^−1^) values using Eq. (6):

6 $$DW = 0.4207\,*\,OD\,750$$

The conversion coefficient of 0.4207 was obtained from the empirical data of the final harvested *H. magna* biomass samples from the cross-gradient experiment. Linear regression was applied to all pairs of OD 750 and DW data to determine the conversion coefficient (Additional file 1: Fig. S6).

#### Linear regression and graph formation

To create the line plots, bar charts, and pie plots, and for simple linear regression calculations, Prism 9 software (GraphPad Software) was used. For the heat plot construction, the program SigmaPlot 14 (Alfasoft AB) was employed.

## Supplementary Information

**Additional file 1 **Additional experiments Methods. Additional experiments Results and discussion. Additional experiments References. **Table S1.** Content and productivity values of biomass (DW) and target products (FX and PUFA) in *H. magna* cultures at the end of the two-phase cultivation experiment. **Table S2.** Composition of WC medium and its modified variants. **Fig. S1.** Important growth parameter characteristics defined by the Weibull growth curve equation in the temperature × light cross-gradient experiment. **Fig. S2.** Time progression of H. magna FX content (mg DWg-1) at different culture conditions in the temperature × light cross-gradient experiment.** Fig S3.** Picture of H. magna culture under the optical microscope. **Fig S4.** Expected and obtained growth of the batch and the two-phase cultivation. **Fig. S5.** Effect of light spectra on *H*.* magna* biomass yield (g L-1) and content (mg DWg-1) of target products. **Fig S6.** Correlation of the absorbance (OD 750) and biomass density (DW).

**Additional file 2 **Growth curves fitted by the Weibull growth curve equation; productivity curves; specific growth rate curves. Calculated for the temperature × light cross-gradient experiment.

**Additional file 3 **Fatty acids profile of final harvested *H. magna *biomass samples of the temperature × light cross-gradient experiment.

## Data Availability

All available data for this study are included in this published article and its supplementary files.

## Abbreviations

DW: Biomass density = dry weight
OD 750: Absorbance at 750 nm wavelength
Prod_mean_: Mean productivity
Prod_max_: Maximal productivity
µ: Specific growth rate
µ_max_: Maximal specific growth rate
FX: Fucoxanthin
FA: Fatty acids
SFA: Saturated fatty acids
MUFA: Monounsaturated fatty acids
PUFA: Polyunsaturated fatty acids
LC-PUFA: Long-chain polyunsaturated fatty acids ≥ 20C
EPA: Eicosapentaenoic acid 20:5 (Ω^−3^)
DHA: Docosahexaenoic acid 22:6 (Ω^−3^)
SDA: Stearidonic acid 18:4 (Ω^−3^)
HPLC: High-performance liquid chromatography
FID: Flame ionization detector
ISTD: Internal standard
