# Supplementary material for: Hibberdia magna (Chrysophyceae): a promising freshwater fucoxanthin and polyunsaturated fatty acid producer
Source: Microb Cell Fact. 2023 Apr 19;22:73. doi: 10.1186/s12934-023-02061-x (PMC10116740; doi:10.1186/s12934-023-02061-x)
Supplement: Supplementary file 1 — Additional file 1 Additional experiments Methods. Additional experiments Results and discussion. Additional experiments References. Table S1. Content and productivity values of biomass (DW) and target products (FX and PUFA) in H. magna cultures at the end of the two-phase cultivation experiment. Table S2. Composition of WC medium and its modified variants. Fig. S1. Important growth parameter characteristics defined by the Weibull growth curve equation in the temperature × light cross-gradient experiment. Fig. S2. Time progression of H. magna FX content (mg DWg-1) at different culture conditions in the temperature × light cross-gradient experiment. Fig S3. Picture of H. magna culture under the optical microscope. Fig S4. Expected and obtained growth of the batch and the two-phase cultivation. Fig. S5. Effect of light spectra on H. magna biomass yield (g L-1) and content (mg DWg-1) of target products. Fig S6. Correlation of the absorbance (OD 750) and biomass density (DW). [file 12934_2023_2061_MOESM1_ESM.pdf]

Additional file 1:

# *Hibberdia magna* (Chrysophyceae): a promising freshwater Fucoxanthin and Polyunsaturated fatty acid producer

## Additional experiments - Methods

### Two-phase cultivation

This experiment was performed in three biological replications cultivated simultaneously. The design of this experiment was based on the preliminary results of the temperature light cross-gradient experiment. The aim was to test the impact of the transition of culture conditions during cultivation to enhance the accumulation and productivity of target products. The experiment was divided into two 168-hour long phases. In the first phase, the culture conditions were the same for all cultures (20°C × 240 µmol $m^{-2}$ $s^{-1}$). This condition was selected to assure fast and stable *H. magna* growth. In the second period, three different experimental setups were tested. In this period the conditions were changed to conditions favorable for (A) FA accumulation (14°C × 480 µmol $m^{-2}$ $s^{-1}$); (B) FX accumulation (23°C × 40 µmol $m^{-2}$ $s^{-1}$); (C) a combination of both (14°C × 40 µmol $m^{-2}$ $s^{-1}$). General culture conditions such as starting density, culture volume, vessel shape, bubbling, etc. were the same as for the cross-gradient experiment (see methods 4.3.1. in the main article). During the first period, only OD 750 measurements were performed on daily basis. After the transition to the second period, the additional samples containing ~5 ml of culture were collected and DW density was determined from 2 mL of the culture the rest of the sample was centrifuged, lyophilized, and kept at -75°C for FX and FA content analysis. These samples were collected at times 0, 6, 12, 24, 48, 72, 120, and 168 hours after the transition of culture conditions.

### Effect of the light spectra

This experiment was performed in three biological replications cultivated simultaneously in the two laboratory-scale culturing units - Multi-cultivator (MC1000-OD-MIX, Photon Systems Instruments). Cultivation was carried out in round bottom cultivation tubes (diameter 25 mm) filled with 80 mL of the starting culture (cell density of approx. 0.5 mil. cells $L^{-1}$) in a batch mode. The temperature was set at 20°C. Cultures were mixed by bubbling with air sterilized by filtration (Filter Sartorius Midisart 2000, 0.2 µm PTFE, Type: 17805) enriched by $CO_2$ to 1% (v/v). The light intensity of 200 µmol $m^{-2}$ $s^{-1}$ was identical for all cultures but the different wavelengths emitting LEDs were used. Five light conditions in total were tested with the following spectral setups: 470 nm (BLUE), 530 nm (GREEN), 660 nm (RED), a 1:1 mixture of 470 nm and 660 nm (PURPLE), and full spectra which was a 1:1:1:1 mixture of blue, green, red, and white LEDs (RGBW). All cultures were harvested at identical times after 170 hours of cultivation and the final DW per volume was determined.

## Additional experiments - Results and discussion

### Two-phase cultivation

The two-phase microalgae cultivation technique is an already established concept utilized by industrial producers of the green alga *Haematococcus pluvialis* for the production of the carotenoid astaxanthin [1]. This idea was applied also to other products, e.g. algal lipids [2,3]. The basic idea of this technique is to divide cultivation into the phase of cell density growth and the phase of a target product accumulation by changing culture conditions. The overall productivity of the target product must be carefully evaluated, and the enhanced accumulation in the second phase must compensate for the decrease in growth rate to make this technique feasible.

The results of the cross-gradient experiment demonstrated that the conditions optimal for biomass productivity and those for the target products accumulation differed. Therefore, we designed a two-stage cultivation procedure, to test the ability of *H. magna* to accumulate the target products in an already-grown culture after the cultivation condition transition. The first growing period lasted for 168 hours and during this period the mean DW productivity was satisfactory 0.32 ± 0.01 g $L^{-1}$ $d^{-1}$ and the cultures reached the mean biomass densities of 2.25 ± 0.06 g $L^{-1}$ (Table S1, this File). After this period, the transition of the culture conditions was performed to enhance FX and PUFA content. Three different transition conditions were tested A) low temperature (LT) (14°C) and low light (LL) (40 µmol $m^{-2}$ $s^{-1}$); B) LT and high light (HL) (480 µmol $m^{-2}$ $s^{-1}$); C) high temperature (HT) (23°C) and LL. The second phase of the experiment lasted for another 168 hours and the DW density, FX, total FA, and PUFA contents are shown in Table 3 for the time zero (transition), three days after transition, and for the final harvested biomass.

Generally, the results of this experiment showed that this two-stage cultivation approach is not feasible for either biomass quality or target compound production rate enhancement. *H. magna* did not cope well with the cultivation condition instability. Immediately after the transition, the culture growth was interrupted, and the culture OD 750 stopped increasing or even decreased in a couple of days (Fig. S4, this File). The most stressful conditions in the second phase of the experiment were the combination of HT and LL. Under these conditions, cultures stopped growing immediately and by the end of the experiment most of the cultures died; no increase in FX content was observed. The cultures under the combination of LT and LL were more stable but the expected increase of target compounds was not observed. The combination of LT and HL brought the best results from these tree transition conditions, even a slight increase in DW density three days after the transition was observed but the quantities and productivities of target compounds (PUFA) were lower than those achieved in the cross-gradient experiment stable optimal condition. In the stable conditions of one stage batch culture technique used

in the previous cross-gradient experiment, the cultures firstly did not interrupt the growth as they did after the transition; secondly, the expected accumulation of the target compound after the transition was not observed. Hence the cultivation in stable conditions brought better results than the two-stage cultivation. We cannot determine clearly whether the transitions during the culture growth should enhance the final productivity of target products, but the transition we tested in the present study was probably too much sudden. Testing a gradual change of the light intensity or temperature alone or changing the light spectra during culture growth may bring a better result in the future.

**Effect of light spectra**

The effect of the light spectra on DW, FX, and FA productivities was examined in a little different experimental setup and thus the resulting productivities and contents slightly differed compared to the cross-gradient results. These differences were caused by the different culture vessel diameters and different harvest times, which were set within the exponential growth phase.

Generally, it was observed that the absence of red light spectra had a negative impact on *H. magna* biomass productivity. The highest DW productivity was obtained at purple light (half to half red and blue LEDs) accounting for the mean productivity of 0.28 ± 0.01 g $L^{-1}$ $d^{-1}$; the lowest DW mean productivity of 0.20 ± 0.01 g $L^{-1}$ $d^{-1}$ was gained at the green light (Fig. S5B, this File). The content of FX was also to a high degree influenced by the light wavelength. The highest total content of FX was observed at the blue light and reached 8.40 ± 0.18 mg $DWg^{-1}$ while the lowest was obtained at red light accounting for 4.02 ± 1.00 mg $DWg^{-1}$ (Fig. S5A, this File). Higher FX content at the blue light was consistent with comparable results obtained for Haptophyceae *Tisochrysis lutea* and diatoms *Thalassiosira weissflogi* and *Cylindrotheca closterium* [4,5,6]. Oppositely, Zhang et al. [7] reported slightly higher FX content at the red light than at the blue light for marine diatom *Odontella aurita*. Even though the blue light showed the highest FX content per DW, the purple light caused higher FX productivity due to the higher biomass growth rate. FX productivity at purple light reached the value of 1.91 ± 0.15 mg $L^{-1}$ $d^{-1}$ (Fig S5B, this File), which was also the highest value achieved by *H. magna* across all experiments.

Total FA content was not strongly influenced by light wavelengths and ranged between the minimum of 100.3 ± 8.7 mg $DWg^{-1}$ at the blue light and the maximum of 126.0 ± 6.1 mg $DWg^{-1}$ at the white light. The content of important Omega3 and Omega6 PUFA was slightly more diverse. The sum of Omega3 PUFA was in a narrow range from 33.6 ± 1.2 to 36.3 ± 2.3 mg $DWg^{-1}$ for all wavelengths except the red light, where the sum content of Omega3 PUFAs was only 21.2 ± 3.5 mg $DWg^{-1}$. In contrast, at red light Omega6 PUFA content was the highest (20.2 ± 1.4 mg $DWg^{-1}$) followed by cultures grown at white; green; purple lights which had similar content (~17.5 mg $DWg^{-1}$), and cultures grown at blue light had the

lowest Omega6 PUFAs total content (10.1 ± 1.4 mg $DWg^{-1}$). Due to these differences, the Omega6 to Omega3 PUFA ratio was significantly influenced by the light spectra (Fig. S5A, this File) varied from 0.28 to 0.95 for the blue and the red light grown cultures, respectively. Due to the highest biomass growth rate of the cultures cultivated under the purple light conditions, these wavelengths appeared to be the best option also for FX productivity as well as for FA (33.4 ± 1.8 mg $L^{-1}$ $d^{-1}$) and PUFA productivity (14.7 ± 1.2 mg $L^{-1}$ $d^{-1}$), but FA productivity values were comparable to those obtained under the white light conditions - the total FA (34.7 ± 1.7 mg $L^{-1}$ $d^{-1}$) and PUFA productivity (15.2 ± 0.9 mg $L^{-1}$ $d^{-1}$).

## Additional experiments - References


[1] Li X, Wang X, Duan C, Yi S, Gao Z, Xiao C, et al. Biotechnological production of astaxanthin from the microalga Haematococcus pluvialis. Biotechnol Adv 2020;43:107602. https://doi.org/10.1016/j.biotechadv.2020.107602.

[2] Remmers IM, Hidalgo-Ulloa A, Brandt BP, Evers WAC, Wijffels RH, Lamers PP. Continuous versus batch production of lipids in the microalgae Acutodesmus obliquus. Bioresour Technol 2017;244:1384–92. https://doi.org/10.1016/j.biortech.2017.04.093.

[3] Zhang D, Xue S, Sun Z, Liang K, Wang L, Zhang Q, et al. Investigation of continuous-batch mode of two-stage culture of Nannochloropsis sp. for lipid production. Bioprocess Biosyst Eng 2014;37:2073–82. https://doi.org/10.1007/s00449-014-1185-6.

[4] Gao F, Woolschot S, Cabanelas ITD, Wijffels RH, Barbosa MJ. Light spectra as triggers for sorting improved strains of Tisochrysis lutea. Bioresour Technol 2021;321. https://doi.org/10.1016/j.biortech.2020.124434.

[5] Marella TK, Tiwari A. Marine diatom Thalassiosira weissflogii based biorefinery for co-production of eicosapentaenoic acid and fucoxanthin. Bioresour Technol 2020;307. https://doi.org/10.1016/j.biortech.2020.123245.

[6] Wang S, Verma SK, Hakeem Said I, Thomsen L, Ullrich MS, Kuhnert N. Changes in the fucoxanthin production and protein profiles in Cylindrotheca closterium in response to blue light-emitting diode light. Microb Cell Fact 2018;17:1–13. https://doi.org/10.1186/s12934-018-0957-0.

[7] Zhang H, Gong P, Cai Q, Zhang C, Gao B. Maximizing fucoxanthin production in Odontella aurita by optimizing the ratio of red and blue light-emitting diodes in an auto-controlled internally illuminated photobioreactor. Bioresour Technol 2022;344:126260. https://doi.org/10.1016/j.biortech.2021.126260.

| | DW | | FX | | Total FA | | Σ PUFA | |
|---|---|---|---|---|---|---|---|---|
| | Quant [g $L^{-1}$] | Prod [g $L^{-1}$ $d^{-1}$] | Quant [mg $DWg^{-1}$] | Prod [mg $L^{-1}$ $d^{-1}$] | Quant [mg $DWg^{-1}$] | Prod [mg $L^{-1}$ $d^{-1}$] | Quant [mg $DWg^{-1}$] | Prod [mg $L^{-1}$ $d^{-1}$] |
| Inok.* | 0.04 | n.a. | 9.85 | n.a. | 116.77 | n.a. | 67.51 | n.a. |
| $Trans_{7Day}$** | 2.25 ± 0.06 | 0.32 ± 0.01 | 3.28 ± 0.10 | 1.00 ± 0.04 | 125.67 ± 12.21 | 39.72 ± 3.71 | 56.71 ± 11.04 | 17.84 ± 3.56 |
| LT_LL $_{10Day}$ | 2.08 ± 0.06 | 0.20 ± 0.01 | 2.91 ± 0.34 | 0.57 ± 0.09 | 121.79 ± 26.22 | 25.01 ± 6.25 | 58.4 ± 16.58 | 11.96 ± 3.84 |
| LT_HL $_{10Day}$ | 2.95 ± 0.08 | 0.29 ± 0.01 | 1.99 ± 0.05 | 0.55 ± 0.03 | 130.51 ± 2.66 | 38.00 ± 1.81 | 49.57 ± 2.49 | 14.33 ± 0.62 |
| HT_LL $_{10Day}$ | 1.65 ± 0.01 | 0.16 ± 0.00 | 3.19 ± 0.21 | 0.49 ± 0.03 | 103.84 ± 8.33 | 16.63 ± 1.31 | 50.14 ± 5.21 | 7.98 ± 0.82 |
| LT_LL $_{14Day}$ | 1.86 ± 0.24 | 0.13 ± 0.02 | 1.91 ± 0.73 | 0.23 ± 0.13 | 92.82 ± 16.06 | 12.18 ± 3.82 | 36.87 ± 12.37 | 4.85 ± 2.34 |
| LT_HL $_{14Day}$ | 2.53 ± 0.16 | 0.18 ± 0.01 | 1.20 ± 0.15 | 0.19 ± 0.04 | 126.52 ± 12.83 | 22.59 ± 3.68 | 45.13 ± 6.78 | 8.00 ± 1.71 |
| HT_LL $_{14Day}$ | 0.67 ± 0.04 | 0.05 ± 0.00 | 1.41 ± 0.24 | 0.04 ± 0.01 | 51.62 ± 6.66 | 2.16 ± 0.44 | 15.13 ± 3.47 | 0.54 ± 0.20 |
| Best FA $_{13.5\ Day}$ | 3.54 ± 0.85 | ~0.26 | 2.11 ± 0.21 | ~0.50 | 195.99 ± 36.63 | **~51.25** | 82.66 ± 17.13 | **~21.52** |
| Best FX $_{13.5\ Day}$ | 2.05 ± 0.05 | ~0.15 | 8.72 ± 0.54 | **~1.27** | 133.26 ± 3.65 | ~19.70 | 62.98 ± 2.23 | ~9.25 |

**Table S1:**

Content (Quant) and productivity values (Prod) of biomass (DW) and target products (FX and PUFA) in *H. magna* cultures at the end of the two-phase cultivation experiment. LT - low temperature (14°C); HT - high temperature (23°C); LL - low light (40 µmol $m^{-2}$ $s^{-1}$); HL - high light (480 µmol $m^{-2}$ $s^{-1}$). * only one sample analyzed (n=1); ** nine cultures in the time of transition analyzed (n=9); rest of the data (n=3). Values are shown as mean values ± standard deviation. Best FA (17°C × 320 µmol $m^{-2}$ $s^{-1}$); Best FX (23°C × 80 µmol $m^{-2}$ $s^{-1}$). The highest values were highlighted in bold.

| **Component** *(Concentration mg/L)* | **WC** *(original)* | **WC+ no Buff** *(maintenance, inoculum)* | **2WC+ 1000MES** *(experiments)* |
|---|---|---|---|
| *Buffer:* | | | |
| Tris | 500 | - | - |
| MES | - | - | 1000 |
| *Macro-nutrients:* | | | |
| $CaCl_2 \cdot 2H_2O$ | 36.8 | 36.8 | 73.6 |
| $MgSO_4 \cdot 7H_2O$ | 37 | 37 | 74 |
| $NaHCO_3$ | 12.6 | 12.6 | 25.2 |
| $NaNO_3$ | 8.7 | 87 | 87 |
| $K_2HPO_4$ | 85 | 850 | 850 |
| $Na_2SiO_3 \cdot 9H_2O$ | 28.4 | 28.4 | 56.8 |
| *Micro-nutrients:* | | | |
| $Na_2EDTA$ | 4.36 | 4.36 | 8.72 |
| $FeCl_3 \cdot 6H_2O$ | 3.15 | 3.15 | 6.3 |
| $H_3BO_3$ | 1 | 1 | 2 |
| $MnCl_2 \cdot 4H_2O$ | 0.18 | 0.18 | 0.36 |
| $ZnSO_4 \cdot 7H_2O$ | 0.022 | 0.022 | 0.044 |
| $CoCl_2 \cdot 6H_2O$ | 0.01 | 0.01 | 0.02 |
| $CuSO_4 \cdot 5H_2O$ | 0.01 | 0.01 | 0.02 |
| $Na_2MoO_4 \cdot 2H_2O$ | 0.006 | 0.006 | 0.012 |
| *Vitamins:* | | | |
| Thiamin HCl (Vit. $B_1$) | 0.1 | 0.1 | 0.1 |
| Biotin (Vit. H) | 0.0005 | 0.0005 | 0.0005 |
| Cyanocobalamin (Vit. $B_{12}$) | 0.0005 | 0.0005 | 0.0005 |

**Table S2:**

Composition of WC medium and its modified variants used for cultivation of *H. magna* K-1175.

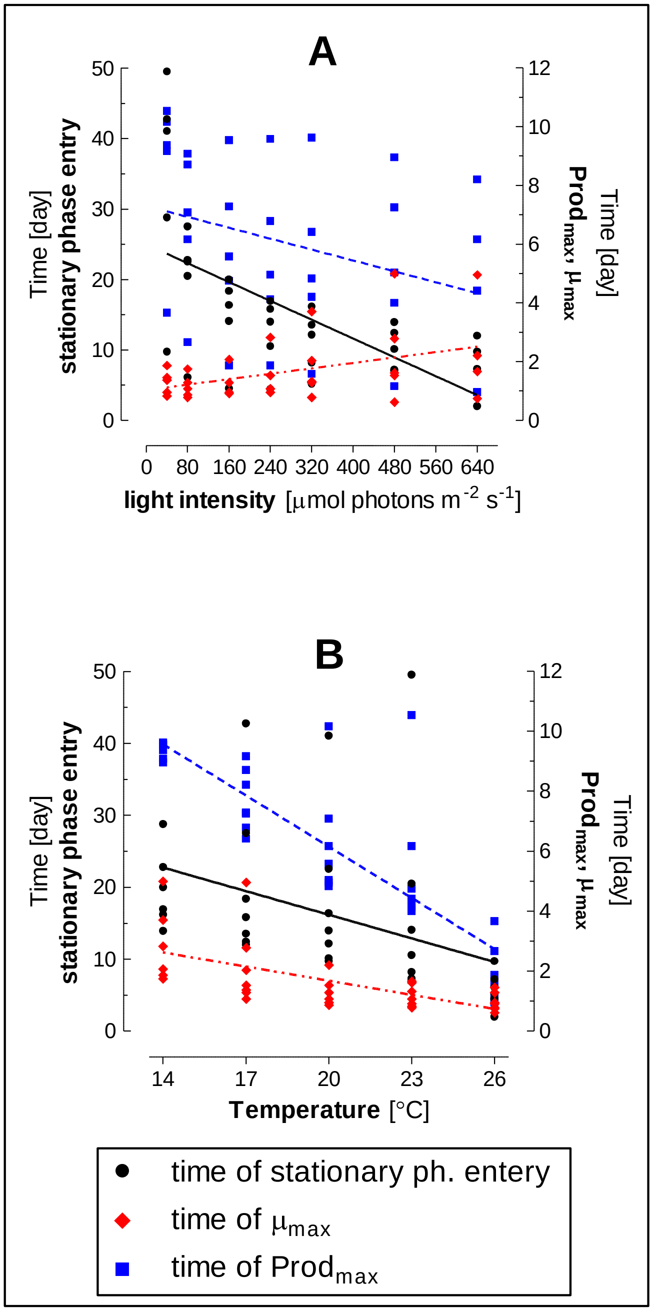


**Fig. S1:** Important growth parameter characteristics (time of stationary ph. Entry; Time of $\mu_{max}$; Time of Prod$_{max}$) as defined by the Weibull growth curve equation in the temperature × light cross-gradient experiment and their dependence on light (A) and temperature (B): The time of stationary phase entry was defined as the time of reaching the density value equal to 99% of the upper asymptote of the Weibull growth curve. The time of $\mu_{max}$ is a time of reaching the maximal specific growth rate. The time of Prod$_{max}$ is a time of reaching the maximal volumetric productivity. Both graphs present the same dataset.

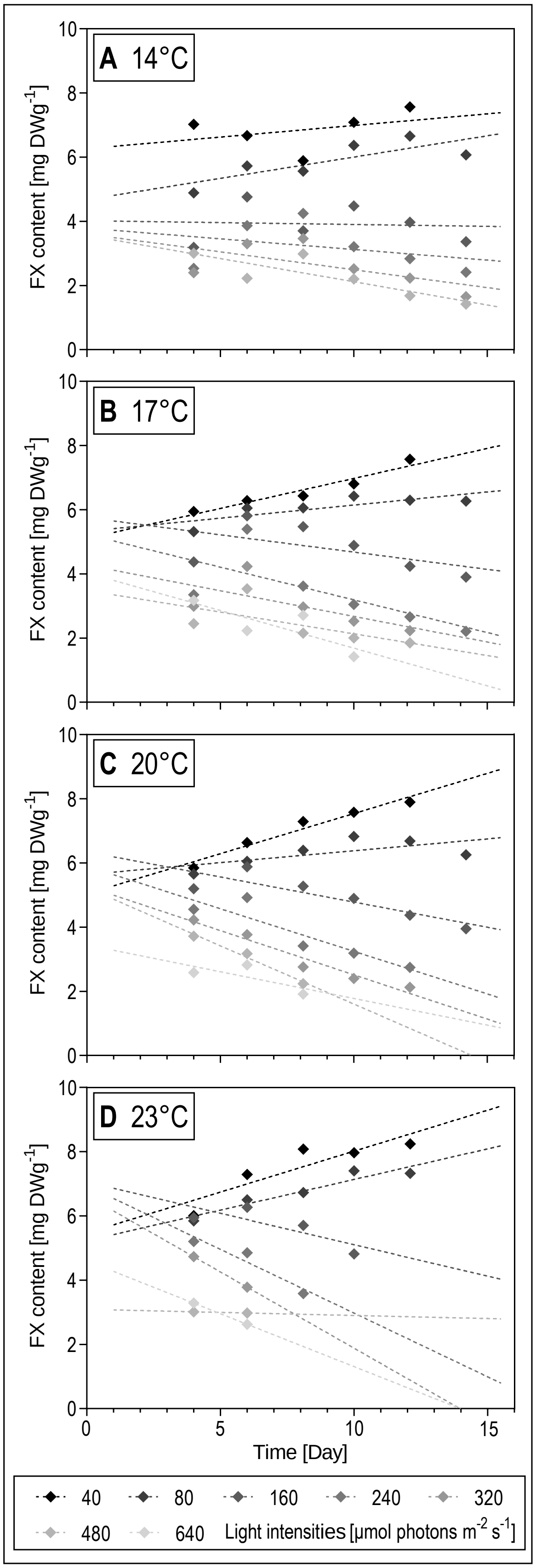


**Fig. S2:** Time progression of *H. magna* Fucoxanthin (FX) content (mg DWg$^{-1}$) at different culture conditions in the temperature × light cross-gradient experiment. Only RUN 1 data was analyzed (n=1).

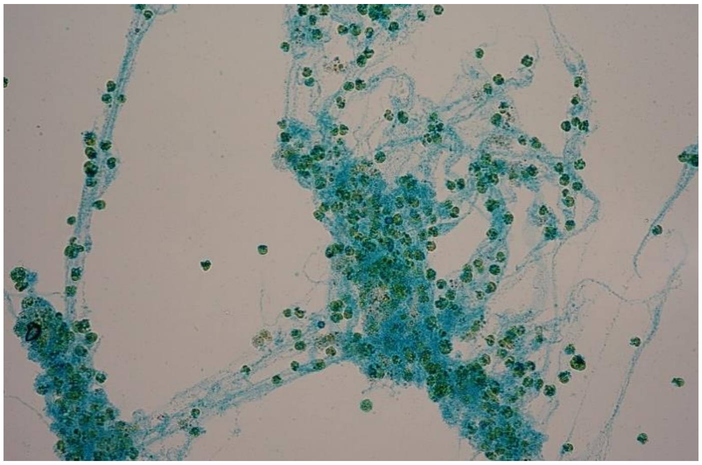

**Fig. S3:** Picture of *H. magna* culture under the optical microscope. Alcian blue stain was used to color the extracellular polysaccharides. Cells are 3 – 5 µm in size.

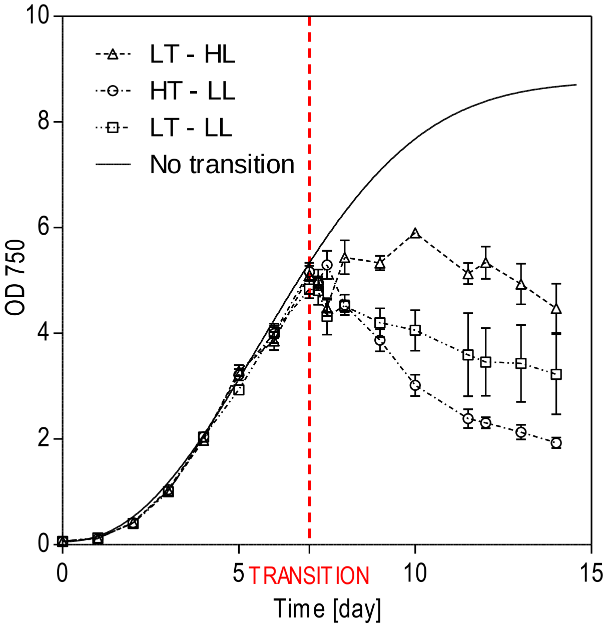


**Fig. S4:** Expected (solid line) and obtained (dashed lines) growth of the batch culture of the cultures at the two-phase cultivation experiment (OD 750): LT - low temperature (14°C); HT - high temperature (23°C); LL - low light (40 µmol $m^{-2}$ $s^{-1}$); HL - high light (480 µmol $m^{-2}$ $s^{-1}$). Symbols are the mean of the three independent replicates (n=3) and SD is indicated.

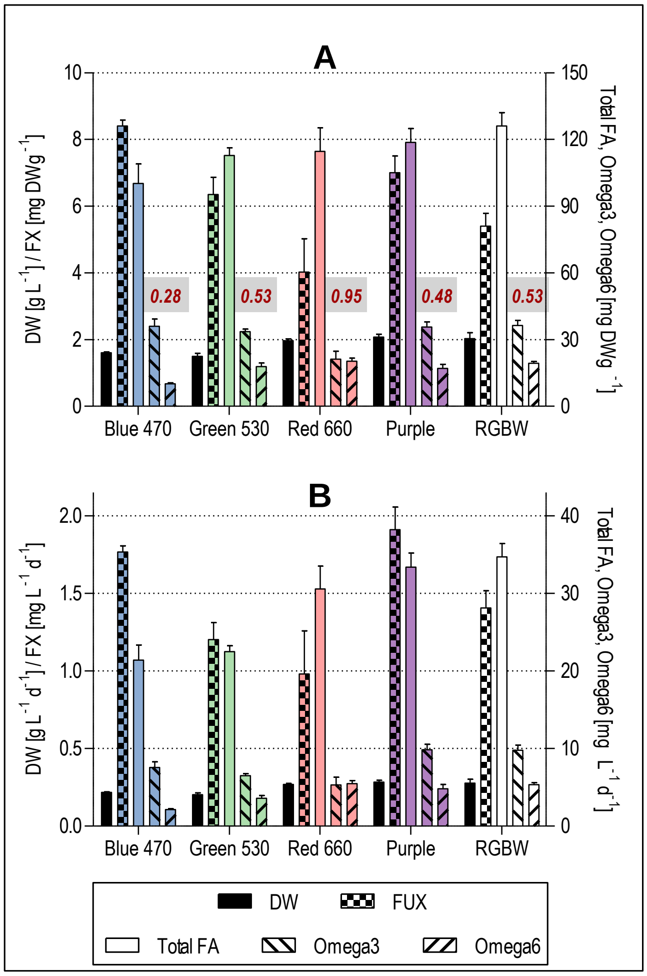


**Fig. S5:** Effect of light spectra on *H. magna* biomass yield (g $L^{-1}$) and content (mg $DWg^{-1}$) of target products (FX; FA; Omega3 PUFA; Omega6 PUFA). The cultures were subjected to light of the same intensity (Purple is a 1:1 mixture of Blue and Red, RGBW is a 1:1:1:1 mixture of Red, Green, Blue, and White): (A) final harvested contents, the red bold number represents the Omega6 : Omega3 PUFA ratio; (B) productivity of biomass (g $L^{-1}$ $Day^{-1}$) and productivity of target products (mg $L^{-1}$ $Day^{-1}$). Bars are the mean of the three independent replicates (n=3) and SD is indicated.

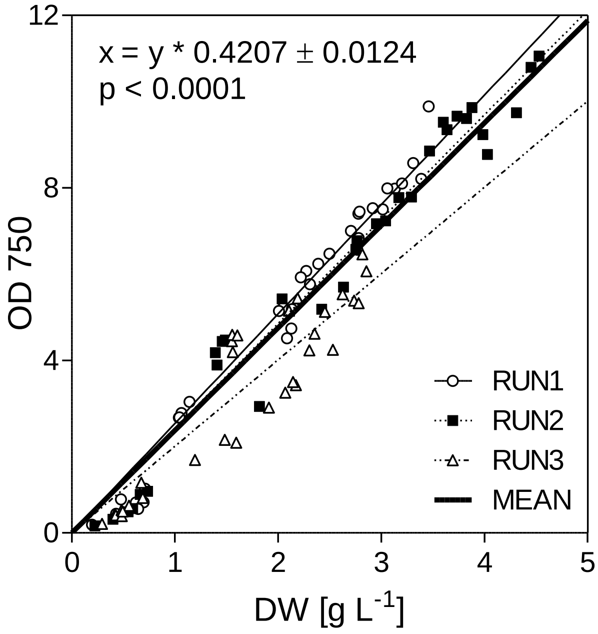


**Fig. S6:** Correlation of the absorbance (OD 750) and biomass density (DW) in *H. magna* cultures. Samples were taken as final harvested samples in the temperature × light cross-gradient experiment. The linear regression was rooted at value 0. Dataset served to determine the OD 750 to DW conversion coefficient used for further calculations.
