# Supplementary material for: Hibberdia magna (Chrysophyceae): a promising freshwater fucoxanthin and polyunsaturated fatty acid producer
Source: Microb Cell Fact. 2023 Apr 19;22:73. doi: 10.1186/s12934-023-02061-x (PMC10116740; doi:10.1186/s12934-023-02061-x)

Additional file 2:

Hibberdia magna (Chrysophyceae): a promising freshwater Fucoxanthin and Polyunsaturated fatty acid producer

Growth curves fitted by the Weibull growth curve equation; productivity curves; specific growth rate curves. Calculated for the temperature × light cross-gradient experiment.

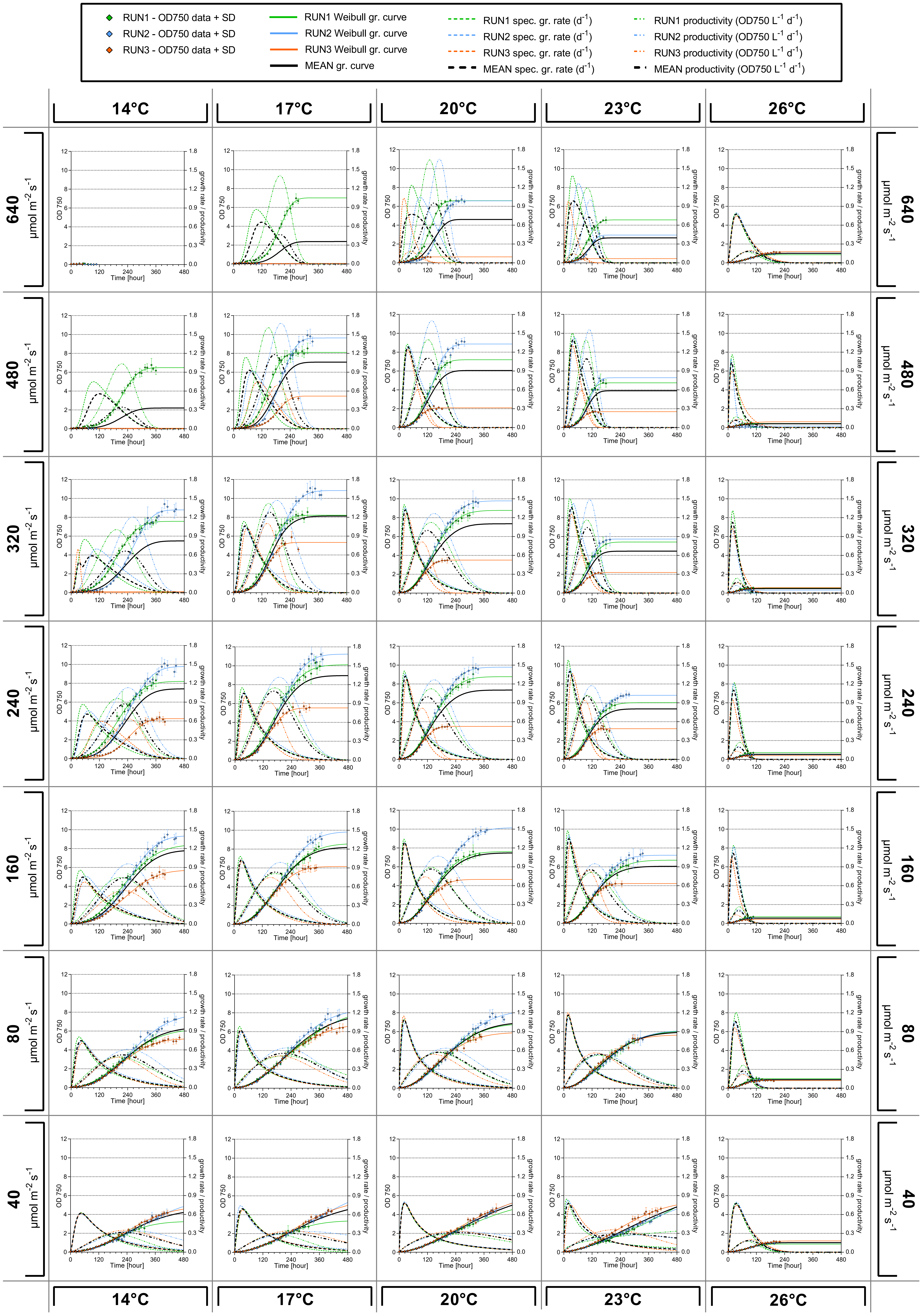

Supplement: Supplementary file 2 — Additional file 2 Growth curves fitted by the Weibull growth curve equation; productivity curves; specific growth rate curves. Calculated for the temperature × light cross-gradient experiment. [file 12934_2023_2061_MOESM2_ESM.pdf]
